# Oxidative Aromatization of 4,7-Dihydro-6-nitroazolo[1,5-a]pyrimidines: Synthetic Possibilities and Limitations, Mechanism of Destruction, and the Theoretical and Experimental Substantiation

**DOI:** 10.3390/molecules26164719

**Published:** 2021-08-04

**Authors:** Daniil N. Lyapustin, Evgeny N. Ulomsky, Ilya A. Balyakin, Alexander V. Shchepochkin, Vladimir L. Rusinov, Oleg N. Chupakhin

**Affiliations:** 1 Department of Organic and Biomolecular Chemistry, Ural Federal University, Mira St. 19, 620002 Ekaterinburg, Russia; lyapustin.danil@yandex.ru (D.N.L.); ulomsky@yandex.ru (E.N.U.); chupakhin@ios.uran.ru (O.N.C.); 2 Institute of Organic Synthesis, Ural Branch of the Russian Academy of Sciences, S. Kovalevskaya Str., 22, 620041 Ekaterinburg, Russia; sasha-mmm@mail.ru; 3 NANOTECH Centre, Ural Federal University, 620002 Ekaterinburg, Russia; i.a.balyakin@gmail.com; 4 Institute of Metallurgy, Ural Branch of the Russian Academy of Sciences, 620016 Ekaterinburg, Russia

**Keywords:** azolo[1,5-a]pyrimidines, nitro-synthons, oxidation, oxidative destruction, synthesis of heterocycles, multicomponent reactions, nitro compounds

## Abstract

The reaction tolerance of the multicomponent process between 3-aminoazoles, 1-morpholino-2-nitroalkenes, and aldehydes was studied. The main patterns of this reaction have been established. Conditions for the oxidation of 4,7-dihydro-6-nitroazolo[1,5-a]pyrimidines were selected. Previous claims that the 4,7-dihydro-6-nitroazolo[1,5-a]pyrimidines could not be aromatised have now been refuted. Compounds with an electron-donor substituent at position seven undergo decomposition during oxidation. The phenomenon was explained based on experimental data, electro-chemical experiment, and quantum-chemical calculation. The mechanism of oxidative degradation has been proposed.

## 1. Introduction

Azolo[1,5-a]pyrimidines are a very diversified group of heterocycles [1,2,3]. Among them, many compounds find application due to their beneficial features, for example, photophysical properties [4,5,6,7], metal complexes [8,9,10], and as medicinal drugs for diabetes mellitus [11,12,13], viruses [14,15,16], bacteria [17,18], neurological diseases [19,20], tumor diseases [21,22,23,24,25,26], Alzheimer’s disease [27,28,29], etc. 

A special place is occupied by 6-nitroazolo[1,5-a]pyrimidines [30]. They are an even more specific class of heterocycles that have been explored much less thoroughly, although due to their nitro group, the variety of their properties is just as wide.

**Figure 1:**
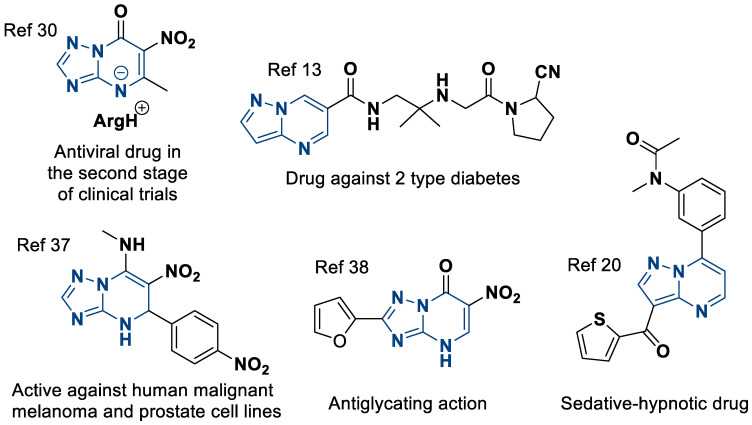
Examples of biologically active azolo[1,5-a]pyrimidines.

From the point of view of biological activity, these compounds exhibit antiviral [31,32], antiseptic [33,34], antioxidant [35,36], antitumor [37], and antiglycation activity [38]. From a synthetic point of view, the nitro groups are not only a quasi-form of the amino groups for creating azoloannelated purines [39,40,41], but they also, for example, open pathways for the modification of molecules by various nucleophiles [42,43]. Some useful azolo[1,5-a]pyrimidines are shown in Figure 1. 

**Scheme 1:**
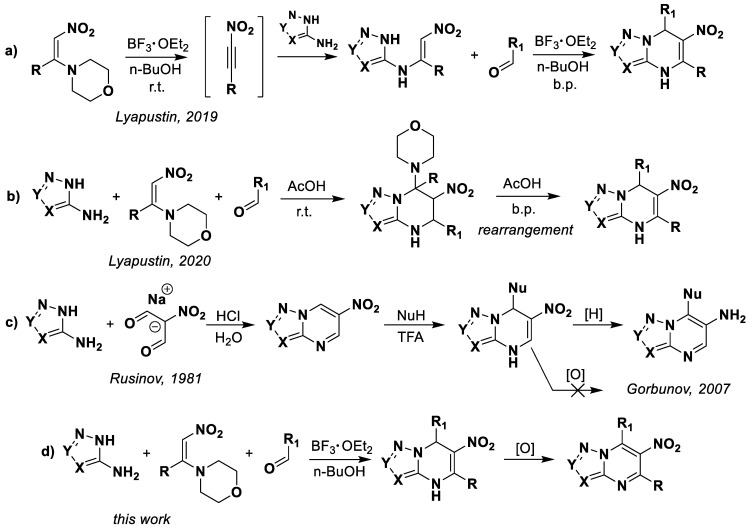
Current methods for the synthesis of 4,7-dihydroazolo[1,5-a]pyrimidines.

Previously we discovered the multicomponent reaction between aminoazoles, aromatic aldehydes, and 1-morpholino-2-nitroalkenes, which proceeds through different mechanisms depending on catalysis with Lewis [44] or Brønsted acids [45] (Scheme 1a,b). Formed that way, 4,7-dihydro-6-nitroazolo[1,5-a]pyrimidines have been obtained by the condensation of corresponding azoles with sodium salts of malonic dialdehyde [46] and the subsequent nucleophilic addition of π-extended (hetero)aromatic systems [43,47,48] (Scheme 1c). However, that approach has the following few disadvantages: a two-step synthesis procedure, an exclusively nucleophilic variant of structural modifications, and the limitation of possible modification positions. Moreover, the inability to aromatize 4,7-dihydro-6-nitroazolo[1,5-a]pyrimidines was established [49]. The method of “reductive autoaromatization” is used for oxidation, where the spontaneous aromatization of the heterocyclic system occurs during the reduction of the nitro group (Scheme 1c).

Thus, in this work we study the possibility of oxidation of 4,7-dihydro-6-nitroazolo[1,5-a]pyrimidines, while saving the nitro-group, and the reaction tolerance of the multicomponent reaction and oxidation process (Scheme 1d). 

## 2. Results

**Scheme 2:**
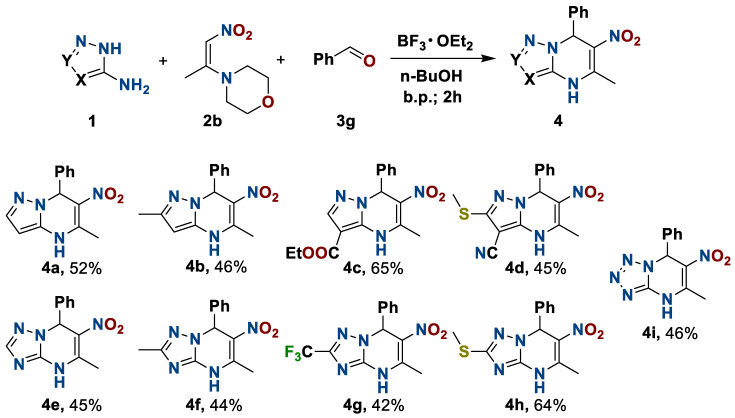
Preparation of 4,7-dihydro-6-nitro-5-methyl-7-phenylazolo[1,5-a]pyrimidines.

To study the substrate scope, we carried out the multicomponent process by changing one of the initial reagents, while two were unchanged. Using 1-morpholino-2-nitropropylene **2b**, benzaldehyde **3g** with varying 3-aminoazoles **1** and molecular fragments in their structure in the medium BF_3_·OEt_2_–n-butanol, we obtained a series of 4,7-dihydro-6-nitro-7-phenylazolo[1,5-*a*]pyrimidines **4** in a moderate to good yield (Scheme 2). 

The next goal of the work was to examine the obtained 4,7-dihydro-5-methyl-6-nitro-7-phenylazolo[1,5-a]pyrimidines **4**. Using compound **4c** as an example, we studied the effect of the nature of solvents and oxidants on the yield of the model reaction product. The results are shown in Table 1. 

We found that the studied compound **4c** decomposes under the action of the strong inorganic oxidizer (Table 1, № 1), and is not oxidized by atmospheric oxygen under acidic conditions (Table 1, № 2), as well as dichlorodicyanobenzoquinone (Table 1, № 3,4), Dess-Martin periodate (Table 1, № 5), and diacetoxyiodobenzene (Table 1, № 7). Nevertheless, the use of pyridinechlorochromate (Table 1, № 6) and diacetoxyiodobenzene (Table 1, № 8) in acetic acid at an elevated temperature leads to the formation of heterocycle **5c** with the yields 49 and 81%, respectively. 

**Scheme 3:**
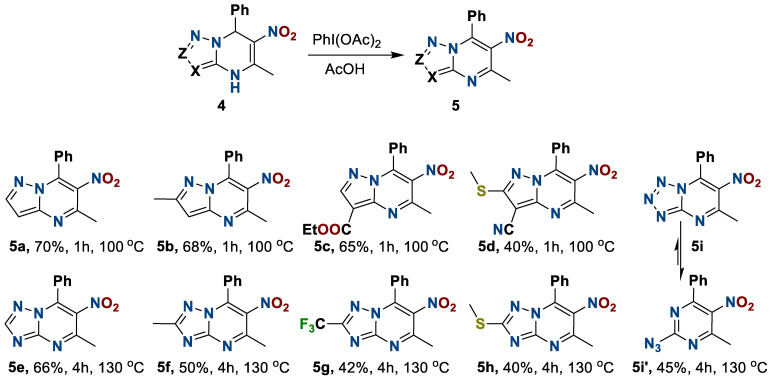
Preparation of 6-nitro-5-methyl-7-phenylazolo[1,5-a]pyrimidines.

Thereby, by using diacetoxyiodobenzene in acetic acid at a corresponding temperature, we obtained a number of new 6-nitro-5-methyl-7-phenylazolo[1,5-a]pyrimidines **5** in moderate to high yields (Scheme 3). 

The oxidation of triazolo- and tetrazolopyrimidines **4e–i** occurs under harsher conditions than the pyrazolo- derivatives **4a–d**; the process requires both a higher temperature and a longer reaction time. This is apparently due to the increasing π-deficient properties of five-membered heterocycles with the increasing numbers of N-atoms. A feature of the tetrazolopyrimidine **5i** is the formation of the azido-tetrazole tautomerism product 2-azido-5-nitro-6-methyl-4-phenylpyrimidine **5i′**, which was supported by IR spectroscopy data (see Supplementary Materials). 

Thus, in the first part of the work, the possibility of the three-component reaction of 1-morpholino-2-nitropropylene **2a**, benzaldehyde **3g**, and 3-aminoazoles **1** was established with an assessment of the last reaction method. In addition, the oxidative aromatization of type **4** dihydroazolopyrimidines, which was previously considered impossible, was realized. 

**Scheme 4:**
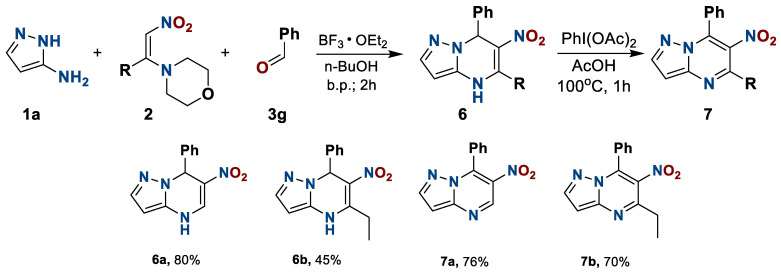
Preparation of 4,7-dihydro-6-nitro-7-phenyl-5-R-pyrazolo[1,5-a]pyrimidines **6** and 4,7-dihydro-6-nitro-7-phenyl-5-R-pyrazolo[1,5-a]pyrimidines **7**.

The next stage of the study was the change the 1-morplholino-2-nitroalkene component **2**. The number of synthetically available nitroalkenes **2** is limited, and all of them turned out to be reactive in the preparation of dihydronitro derivatives **6**, which, in turn, were successfully dehydrogenated (Scheme 4).

**Scheme 5:**
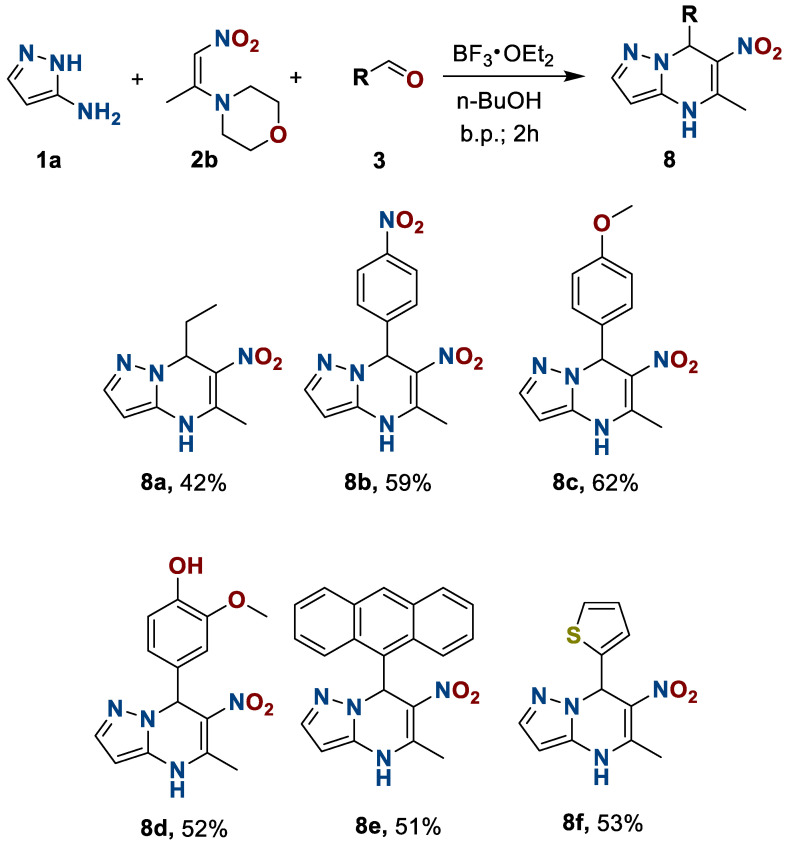
Preparation of 4,7-dihydro-6-nitro-5-methyl-7-R-azolo[1,5-a]pyrimidines **8**.

Finally, we examined the effect of the structure of the third component, aldehydes, on the formation of 4,7-dihydro-6-nitroazolo[1,5-a]pyrimidines **8** and their subsequent oxidation. The object of study was to use benzaldehyde **3g** and its derivatives with electron-donating **3c,d** and electron-acceptor **3b** groups, polycyclic anthracenecarbaldehyde **3e**, heterocyclic thophenecarbaldehyde **3f**, and propanal **3a**. The use of the developed reaction conditions allowed us to obtain 7-R-5-methyl-6-nitropyrazolo[1,5-a]pyrimidines **8** in moderate to good yields (Scheme 5).

**Scheme 6:**
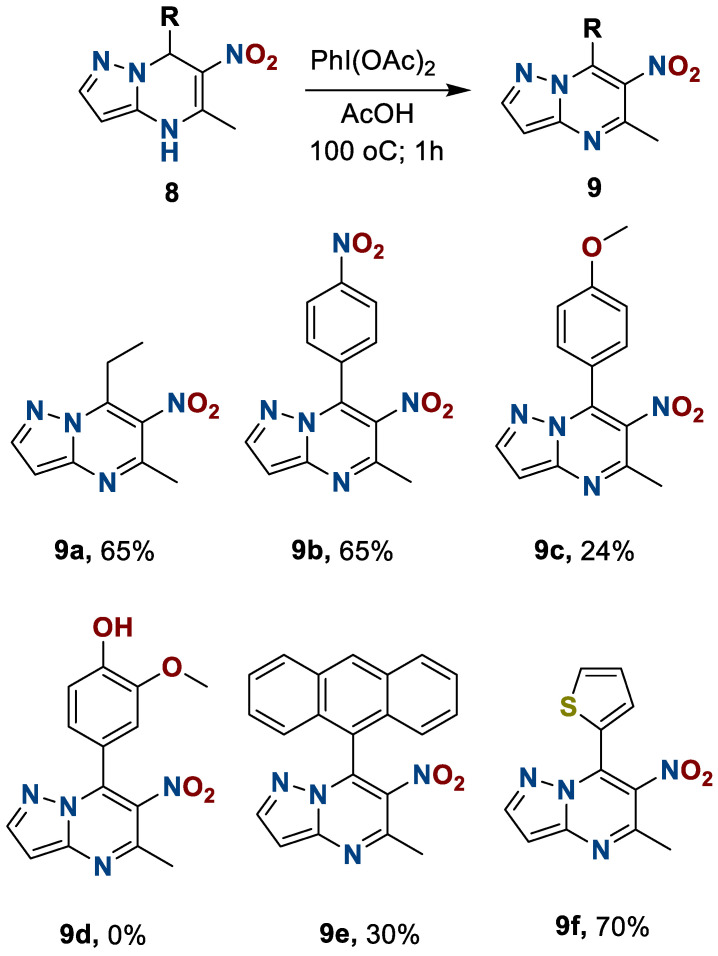
Preparation of 6-nitro-5-methyl-7-R-azolo[1,5-a]pyrimidines **9**.

During the oxidation, we discovered that the interaction of heterocycle **8d** with phenyliodozodiacetate, even at room temperature, leads to the resinification of the reaction mass. At the same time, the oxidation of heterocycles **8c** and **8e** was also accompanied by the formation of side products, but we have succeeded in isolating the desired compounds in yields of 24 and 30%, respectively. The results are presented in Scheme 6. 

**Scheme 7:**
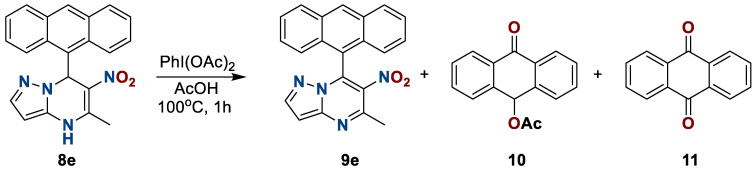
Oxidation of 6-nitro-5-methyl-7-(anthracen-9-yl)azolo[1,5-a]pyrimidine **8e**.

It was previously noted that it is impossible to obtain an aromatic system for similar structures, since attempts at oxidation have led either to unsuccessful results or to a mixture of unidentifiable products. However, results of this work complement the existing precedent since oxidation is complicated only in the case of compounds with an electron-donor substituent at position seven. During the oxidation of nitropyrazolopyrimdines **9e**, besides the main product **9e**, side products 9-acetoxy-anthracene **10** and anthraquinone **11** were also isolated using the flash chromatography method. These compounds were characterized in the composition of the mixture (Scheme 7). 

**Figure 2:**
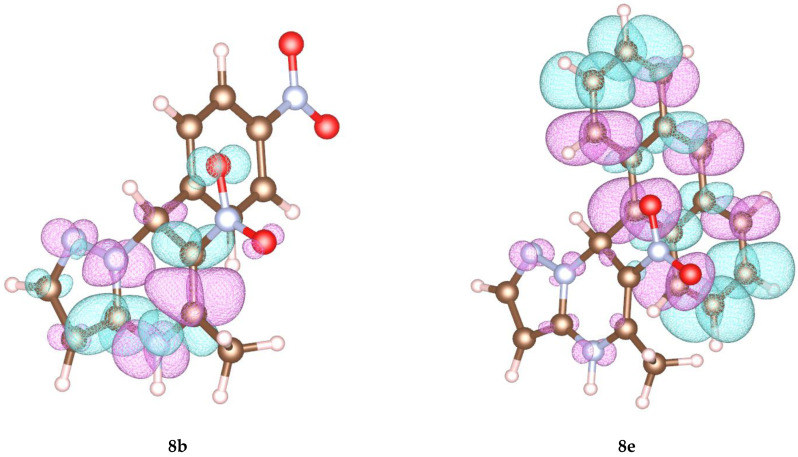
Visualization of HOMO for compounds **8b** and **8e** obtained in PBE0 approximation.

To elucidate the experimental data, we performed a quantum chemical calculation of the compounds **5a, 8a–e** to obtain the charge distribution over the molecular structure. Figure 2 demonstrates the spin distribution of the highest occupied molecular orbital (HOMO) for compounds **8b** and **8e**. Other data are shown in the Supplementary Materials. 

Indeed, the calculated data demonstrate that in compounds with an electron donating substituent **8e**, the substituent is involved in the electron density distribution, which can have a serious influence on the direction of oxidation. Therefore, in these structures, the covalent bond between the C-7 atom and the substituent fissions and the nonstoichiometric formation of oxidation side products occurs.

**Figure 3:**
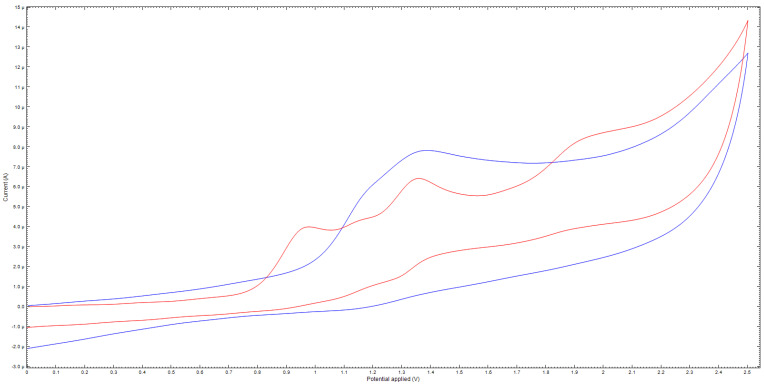
Cyclic voltammogram of **8b** (blue) and **8e** (red), 5 × 10^−3^ M.

Furthermore, the results of cyclic voltammetry also indicate the different behavior of compound **8** under oxidation conditions (Figure 3). Thus, for compound **8b**, one irreversible peak of two-electron oxidation is observed. In turn, compound **8e** is characterized by two peaks, apparently, of one-electron oxidation. The data for other compounds are given in the Supplementary Materials. 

**Scheme 8:**
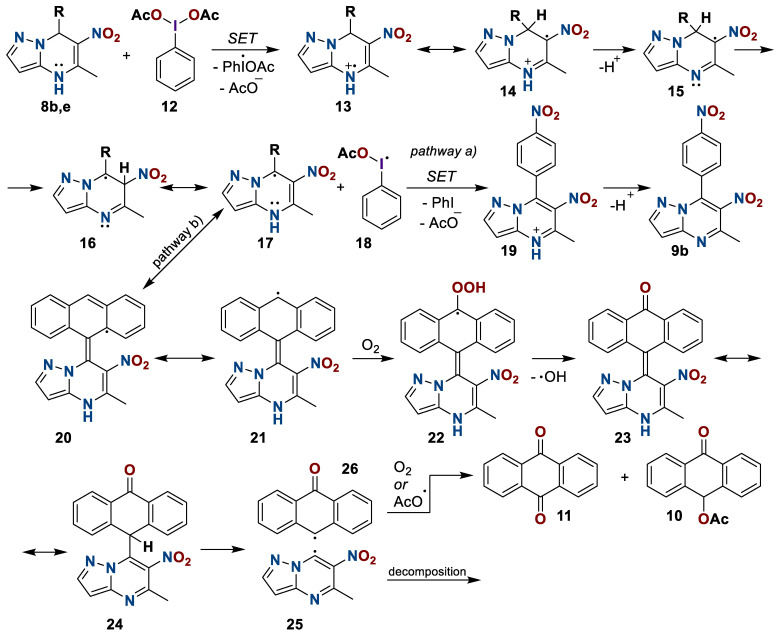
Plausible mechanism of oxidation of 6-nitro-5-methyl-7-R-azolo[1,5-a]pyrimidines **8**.

Based on the results above and the literature’s data [50,51,52], it can be assumed that initially, for both compounds **8b** and **8e**, single electron transfer (SET) occurs with the formation of an acetate-anion and a heterocyclic cation-radical **13** (Scheme 8). Then, the tautomeric transformation, the deprotonation of structure 14, and a shift of the hydrogen radical with the formation of radical particle **15** occur. Next, depending on the nature of the substituent, oxidation proceeds either with one more single electron transfer for compounds with an electron withdrawing substituent at position seven, or with the migration of a radical to a substituent for compounds with an electron donating substituent. Apparently, the intermediate **21** interacts with an oxygen molecule with the formation of carbonyl compound **23**. Further intramolecular transformations lead to the homolytic fission of the covalent bond between the C-7 atom and the substituent in structure **24**. The aromatic radical is either oxidized to anthraquinone 11 or is attacked by the acetoxy-radical from phenyliodozomonoacetate 18. The heterocyclic radical **25**, apparently, decomposes nonstoichometrically.

## 3. Materials and Methods

Unless stated otherwise, all solvents and commercially available reactants/reagents were used as received. Non-commercial starting materials were prepared as described below or according to the literature’s procedures. One-dimensional ^1^H and ^13^C NMR spectra, as well as two-dimensional ^1^H–^13^C HMBC experiments were acquired on a Bruker DRX-400 instrument (400 and 101 MHz, respectively) or a Bruker Avance NEO 600 instrument (600 and 151 MHz, respectively), equipped with a Prodigy broadband gradient cryoprobe, utilizing DMSO-*d*_6_ and CDCl_3_ as the solvent and TMS as the internal standard. IR spectra were recorder on a Bruker Alpha FTIR spectrometer equipped with a ZnSe ATR accessory. Mass spectra were recorded on a Shimadzu GCMS-QP2010 Ultra mass spectrometer, using EI method of ionization (70 eV). Elemental analysis was performed on a PerkinElmer 2400 CHN analyzer. The reaction progress was controlled by TLC on Silufol UV-254 plates, eluent—CH_3_Cl. Melting points were determined on a Stuart SMP3 apparatus at the heating rate of 25 °C/min. 1-Morpholino-2-nitroethylenes **2** were prepared according to a literature procedure [53]. 

Density functional theory (DFT) simulation via Vienna Ab initio Simulation Package (VASP) [54] was performed for structural optimization of the molecules and obtaining of the charge distribution. The length of the supercell of every molecule was set to 22.5 Å and the cut-off energy of plane wave basis set was set to 750 eV. Structural optimization was performed using generalized gradient approximation (GGA) by Pedrew, Burke, and Ernzerhof (PBE) [55] for exchange-correlation potential. For all chemical species, projected augmented wave (PAW) pseudopotentials were used. The structures were optimized using two algorithms: conjugate gradient algorithm was implemented for initial optimization and after that RMM-DIIS (residual minimization scheme, direct inversion in the iterative subspace) algorithm was used for final optimization. The stopping criteria for optimization was |*F_i_*| ≤ 0.01 eV/Å, where *i = x, y, z,* i.e., absolute value of each force component acting on nuclei should be not greater than 10 meV/Å.

For PBE-optimized structures, a hybrid PBE0 simulation was performed to obtain charge distribution, for accounting of long-range interactions Grimme DFT-D3 corrections [56] were used. Since all the performed calculation were spin polarized, it was possible to extract spin density from the charge distribution. For the interpretation of the obtained data, the program VESTA [57] was used.

Cyclic voltammetry was carried out on a Metrohm Autolab PGSTAT**128N** potentiostat with a standard three-electrode configuration. Typically, a three electrodes cell equipped with a glass carbon working electrode, an Ag/AgNO_3_ (0.01 M) reference electrode, and a glass carbon rod counter electrode was employed. The measurements were performed in acetonitrile with tetrabutylammonium hexafluorophosphate (0.1 M) as the supporting electrolyte under an argon atmosphere at a scan rate of 100 mV/s. 

### 3.1. 4,7-dihydro-6-nitroazolo[1,5-a]pyrimidines 4,6,8; General procedure 1

To a suspension 2 mmol (1.0 equiv.) of corresponding aminoazole **1**, 2 mmol (1.0 equiv.) of nitroalkene **2,** and 2 mmol (1.0 equiv.) of aldehyde **3** in 5 mL of n-BuOH, was added 3 mmol (1.5 equiv., 0.37 mL) of BF_3_·Et_2_O. The reaction mixture was heated on oil bath at 120 °C for 2 h. The resulting solution was cooled to room temperature and stirred for 15 min. The obtained precipitate was filtered off, washed with 15 mL of *i*-PrOH. The precipitate was suspended in 50 mL of water, stirred for 5 min, filtered off again, and washed with 15 mL of water.

*5-Methyl-6-nitro-7-phenyl-4,7-dihydropyrazolo[1,5-a]pyrimidine***(4a)**. The reaction was performed according to the general procedure 1, employing 0.166 g (2 mmol, 1 equiv.) of 3-aminopyrazole **1a**, 0.344 g (2 mmol, 1 equiv.) of 1-morpholino-2-nitropropylene **2b** and 0.20 mL (2 mmol, 1 equiv.) of benzaldehyde **3g**. Yellow solid. Yield, 0.266 g (52%); mp, 244–247 °C. IR (ATR): 1516, 1415 (NO_2_). ^1^H NMR (400 MHz, DMSO-*d*_6_): δ = 2.63 (3H, s, CH_3_); 5.48 (1H, s, H-7); 7.09–7.28 (5H, m, Ph); 7.41 (1H, s, H-2); 10.74 (1H, s, NH); 12.37 (1H, s, H-3). ^13^C {^1^H} NMR (101 MHz, DMSO-*d*_6_): δ = 21.9; 39.5; 106.8; 122.8; 126.0; 126.3; 128.3; 126.5; 139.0; 144.4; 146.7; 151.6. Anal. Calcd for C_13_H_12_N_4_O_2_: C, 60.93; H, 4.72; N, 21.86. Found: C, 60.80; H, 4.65; N, 21.90.

*2,5-Dimethyl-6-nitro-7-phenyl-4,7-dihydropyrazolo[1,5-a]pyrimidine***(4b)**. The reaction was performed according to the general procedure 1, employing 0.194 g (2 mmol, 1 equiv.) of 3-amino-5-methylpyrazole **1b**, 0.344 g (2 mmol, 1 equiv.) of 1-morpholino-2-nitropropylene **2b**, and 0.20 mL (2 mmol, 1 equiv.) of benzaldehyde **3g**. Yellow solid. Yield, 0.221 g (46%); mp, 250–255 °C. IR (ATR): 1514, 1417 (NO_2_). ^1^H NMR (400 MHz, DMSO-*d*_6_): δ = 1.92 (3H, s, C-2-CH_3_); 2.60 (3H, s, C-5-CH_3_); 5.36 (1H, s, H-7); 7.06–7.31 (5H, m, Ph); 10.66 (1H, s, NH); 12.07 (1H, s, H-3). ^13^C {^1^H} NMR (101 MHz, DMSO-*d*_6_): δ = 9.4; 21.9; 39.5; 104.0; 123.7; 126.0; 127.0; 128.1; 135.5; 144.7; 145.8; 151.2. Anal. Calcd for C_14_H_14_N_4_O_2_: C, 62.21; H, 5.22; N, 20.73. Found: C, 62.28; H, 5.26; N, 20.65.

*3-Etoxycarbonyl-5-methyl-6-nitro-7-phenyl-4,7-dihydropyrazolo[1,5-a]pyrimidine***(4c)**. The reaction was performed according to the general procedure 1, employing 0.310 g (2 mmol, 1 equiv.) of 3-amino-4-etoxycarbonylpyrazole **1c**, 0.344 g (2 mmol, 1 equiv.) of 1-morpholino-2-nitropropylene **2b**, and 0.20 mL (2 mmol, 1 equiv.) of benzaldehyde **3g**. Bright yellow solid. Yield, 0.426 g (65%); mp, 215–218 °C. IR (ATR): 1685 (C=O); 1576, 1300 (NO_2_). ^1^H NMR (400 MHz, DMSO-*d*_6_): δ = 1.32 (3H, t, CH_2_-CH_3_, *J =* 7.1 Hz); 2.79 (3H, s, C-5-CH_3_); 4.27 (2H, dd, CH_2_-CH_3_, *J =* 7.0, 2.5 Hz); 6.56 (1H, s, H-7); 7.23–7.38 (5H, m, Ph); 7.66 (1H, s, H-2); 10.29 (1H, s, NH). ^13^C {^1^H} NMR (101 MHz, DMSO-*d*_6_): δ = 14.3; 19.8; 59.5; 59.8; 97.5; 122.6; 127.2; 128.4; 128.6; 137.4; 139.6; 140.8; 148.1; 161.7. Anal. Calcd for C_16_H_16_N_4_O_4_: C, 58.53; H, 4.91; N, 17.06. Found: C, 58.41; H, 4.81; N, 17.19.

*3-Cyano-5-methyl-2-methylthio-6-nitro-7-phenyl-4,7-dihydropyrazolo[1,5-a]*pyrimidine **(4d)**. The reaction was performed according to the general procedure 1, employing 0.308 g (2 mmol, 1 equiv.) of 3-amino-4-cyano-5-methylthiopyrazole **1d**, 0.344 g (2 mmol, 1 equiv.) of 1-morpholino-2-nitropropylene **2b**, and 0.20 mL (2 mmol, 1 equiv.) of benzaldehyde **3g**. Sand color solid. Yield, 0.294 g (45%); mp, 248–256 °C with decomp. IR (ATR): 2229 (CN); 1575, 1306 (NO_2_). ^1^H NMR (400 MHz, DMSO-*d*_6_): δ = 2.42 (3H, s, S-CH_3_); 2.67 (3H, s, C-5-CH_3_); 6.53 (1H, s, H-7); 7.27–7.47 (5H, m, Ph); 11.90 (1H, s, NH). ^13^C {^1^H} NMR (101 MHz, DMSO-*d*_6_): δ = 13.8; 19.6; 59.8; 75.3; 112.1; 122.6; 127.4 (2C); 128.6; 139.0; 140.4; 147.4; 150.9. Anal. Calcd for C_15_H_13_N_5_O_2_S: C, 55.04; H, 4.00; N, 21.39. Found: C, 55.00; H, 4.04; N, 21.50.

*5-Methyl-6-nitro-7-phenyl-4,7-dihydro-1,2,4-triazolo[1,5-a]pyrimidine***(4e)**. The reaction was performed according to the general procedure 1, employing 0.168 g (2 mmol, 1 equiv.) of 3-amino-1,2,4-triazole **1e**, 0.344 g (2 mmol, 1 equiv.) of 1-morpholino-2-nitropropylene **2b**, and 0.20 mL (2 mmol, 1 equiv.) of benzaldehyde **3g**. Light yellow solid. Yield, 0.231 g (45%); mp, 270 °C with decomp. IR (ATR): 1582, 1312 (NO_2_). ^1^H NMR (400 MHz, DMSO-*d*_6_): δ = 2.66 (3H, s, CH_3_); 6.63 (1H, s, H-7); 7.26–7.40 (5H, m, Ph); 7.78 (1H, s, H-2); 11.87 (1H, s, NH). ^13^C {^1^H} NMR (101 MHz, DMSO-*d*_6_): δ = 20.2; 59.7; 121.9; 127.3; 128.5; 128.5; 139.3; 145.5; 149.3; 150.9. Anal. Calcd for C_12_H_11_N_5_O_2_: C, 56.03; H, 4.31; N, 27.22. Found: C, 56.10; H, 4.26; N, 27.27.

*2,5-Dimethyl-6-nitro-7-phenyl-4,7-dihydro-1,2,4-triazolo[1,5-a]pyrimidin**e*****(4f)**. The reaction was performed according to the general procedure 1, employing 0.196 g (2 mmol, 1 equiv.) of 3-amino-5-methyl-1,2,4-triazole **1f**, 0.344 g (2 mmol, 1 equiv.) of 1-morpholino-2-nitropropylene **2b**, and 0.20 mL (2 mmol, 1 equiv.) of benzaldehyde **3g**. Yellow solid. Yield, 0.238 g (44%); mp, 270 °C with decomp. IR (ATR): 1586, 1314 (NO_2_). ^1^H NMR (400 MHz, DMSO-*d*_6_): δ = 2.11 (3H, s, C-2-CH_3_), 2.64 (3H, s, C-5-CH_3_); 6.52 (1H, s, H-7); 7.26–7.38 (5H, m, Ph); 11.74 (1H, s, NH). ^13^C {^1^H} NMR (101 MHz, DMSO-*d*_6_): δ = 13.9; 20.2; 59.5; 121.9; 127.3; 128.4; 128.5; 139.5; 145.5; 149.1; 159.3. Anal. Calcd for C_13_H_13_N_5_O_2_: C, 57.56; H, 4.83; N, 25.82. Found: C, 57.49; H, 4.90; N, 25.75.

*5-Methyl-6-nitro-7-phenyl-2-trifluoromethyl-4,7-dihydro-1,2,4-triazolo[1,5-a]pyrimidine***(4g)**.

To a suspension of 0.304 g (2 mmol, 1 equiv.) of 3-amino-5-trifluoromethyl-1,2,4-triazole **1g**, 0.344 g (2 mmol, 1 equiv.) of 1-morpholino-2-nitropropylene **2b** and 0.20 mL (2 mmol, 1 equiv.) of benzaldehyde **3g** in 5 mL of n-BuOH was added 3 mmol (1.5 equiv., 0.37 mL) of BF_3_·Et_2_O. The reaction mixture was heated on oil bath at 120 °C for 2 h. The resulting solution was cooled to room temperature, and 5 mL of n-heptane was added. The obtained suspension was stirred for 10 min, filtered off, and washed with 20 mL of water. The product was recrystallized from *i-*PrOH-H_2_O 1/1. Yellow solid. Yield, 0.273 g (42%); mp, 231–234 °C. IR (ATR): 1573, 1321 (NO_2_). ^1^H NMR (400 MHz, DMSO-*d*_6_): δ = 2.66 (3H, s, C-5-CH_3_); 6.74 (1H, s, H-7); 7.31–7.45 (5H, m, Ph); 12.13 (1H, s, NH). ^13^C {^1^H} NMR (101 MHz, DMSO-*d*_6_): δ = 20.1; 60.4; 118.9 (q, *J =* 269.7 Hz); 122.5; 127.6; 128.7; 128.9; 138.4; 147.0; 148.8; 151.1 (q, *J =* 39.1 Hz). Anal. Calcd for C_13_H_10_F_3_N_5_O_2_: C, 48.01; H, 3.10; N, 21.53. Found: C, 48.15; H, 3.24; N, 21.40.

*5-Methyl-2-methylthio-6-nitro-7-phenyl-4,7-dihydro-1,2,4-triazolo[1,5-a]pyrimidine***(4h)**. The reaction was performed according to the general procedure 1, employing 0.260 g (2 mmol, 1 equiv.) of 3-amino-5-methylthio-1,2,4-triazole **1h**, 0.344 g (2 mmol, 1 equiv.) of 1-morpholino-2-nitropropylene **2b**, and 0.20 mL (2 mmol, 1 equiv.) of benzaldehyde **3g**. Pale yellow solid. Yield, 0.388 g (64%); mp, 273–276 °C. IR (ATR): 1557, 1320 (NO_2_). ^1^H NMR (400 MHz, DMSO-*d*_6_): δ = 2.42 (3H, s, S-CH_3_); 2.64 (3H, s, C-5-CH_3_); 6.56 (1H, s, H-7); 7.27–7.38 (5H, m, Ph); 11.90 (1H, s, NH). ^13^C {^1^H} NMR (101 MHz, DMSO-*d*_6_): δ = 13.5; 20.2; 59.8; 122.2; 127.4; 128.5; 128.6; 139.1; 146.2; 148.8; 160.0. Anal. Calcd for C_13_H_13_N_5_O_2_S: C, 51.49; H, 4.41; N, 23.02. Found: C, 51.47; H, 4.32; N, 23.09. 

*5-Methyl-6-nitro-7-phenyl-4,7-dihydrotetrazolo[1,5-a]pyrimidine***(4i)**. The reaction was performed according to the general procedure 1, employing 0.206 g (2 mmol, 1 equiv.) of 3-aminotetrazole monohydrate **1i**, 0.344 g (2 mmol, 1 equiv.) of 1-morpholino-2-nitropropylene **2b**, and 0.20 mL (2 mmol, 1 equiv.) of benzaldehyde **3g**. To a suspension of 0.206 g (2 mmol, 1 equiv.) of 3-aminotetrazole monohydrate **1i**, 0.344 g (2 mmol, 1 equiv.) of 1-morpholino-2-nitropropylene **2b** and 0.20 mL (2 mmol, 1 equiv.) of benzaldehyde **3g** in 5 mL of n-BuOH was added 3 mmol (1.5 equiv., 0.37 mL) of BF_3_·Et_2_O. The reaction mixture was heated on oil bath at 120 °C for 2 h. The resulting solution was cooled to room temperature, and 5 mL of n-heptane was added. The obtained suspension was stirred for 10 min, decanted, and 5 mL of *i-*PrOH was added. The obtained suspension was filtered off and washed with 20 mL of water. The product was recrystallized from *i-*PrOH. White yellow solid. Yield, 0.237 g (46%); mp, 236–239 °C IR (ATR): 1576, 1295 (NO_2_). ^1^H NMR (400 MHz, DMSO-*d*_6_): δ = 2.68 (3H, s, CH_3_); 7.02 (1H, s, H-7); 7.30–7.50 (5H, m, Ph); 12.30 (1H, s, NH). ^13^C {^1^H} NMR (101 MHz, DMSO-*d*_6_): δ = 20.2; 58.9; 122.2; 127.5; 128.9; 129.1; 138.2; 147.4; 149.4. Anal. Calcd for C_11_H_10_N_6_O_2_: C, 51.16; H, 3.90; N, 32.54. Found: C, 51.10; H, 3.95; N, 32.51.

### 3.2. 6-Nitroazolo[1,5-a]pyrimidines 5,7,9; General procedure 2

To a suspension of 100 mg of corresponding heterocycle **4,6,8** in 5 mL of acetic acid, 1.25 equiv. of PhI(OAc)_2_ was added. The reaction mixture was heated on oil bath at 100 °C for pyrazolopyrimidines and 130 °C for tetra- and triazolopyrimidines for 1.5 h and 4 h, respectively. To the resulting solution, another 0.5 equiv. of PhI(OAc)_2_ was added and stirred for 30 min. After this, 5 mL of EtOH was added and stirred again for 15 min. After heating, the resulting solution was concentrated under reduced pressure. To the residue, 3 mL of n-heptane was added and evaporated again. The residue was purified using flash-chromatography on silica gel 60 with CHCl_3_ as the eluent to give the desirable product. 

*5-Methyl-6-nitro-7-phenylpyrazolo[1,5-a]pyrimidine***(5a)**. The reaction was performed according to the general procedure 2, employing 0.39 mmol of heterocycle **4a** and 0.157 + 0.063 g (0.48 + 0.19 mmol) of PhI(OAc)_2_. Sand color solid. Yield, 69 mg (70%); mp, 232–235 °C. IR (ATR): 1527, 1364 (NO_2_). ^1^H NMR (400 MHz, CDCl_3_): δ = 2.80 (3H, s, CH_3_); 7.48–7.62 (5H, m, Ph); 8.03 (1H, s, H-2); 12.05 (1H, s, H-3). ^13^C {^1^H} NMR (101 MHz, CDCl_3_): δ = 21.8; 113,3; 128,4; 129.7; 130.7; 132.3; 135.7; 138.2; 142.8; 150.6; 151.5. Anal. Calcd for C_13_H_10_N_4_O_2_: C, 61.41; H, 3.96; N, 22.04. Found: C, 61.44; H, 3.93; N, 22.00.

*2,5-Dimethyl-6-nitro-7-phenylpyrazolo[1,5-a]pyrimidine***(5b)**. The reaction was performed according to the general procedure 2, employing 0.39 mmol of heterocycle **4a** and 0.150 + 0.060 g (0.47 + 0.19 mmol) of PhI(OAc)_2_. Sand color solid. Yield, 69 mg (55%); mp, 199–212 °C. IR (ATR): 1525, 1367 (NO_2_). ^1^H NMR (400 MHz, CDCl_3_): δ = 2.04 (3H, s, C-2-CH_3_); 2.77 (3H, s, C-5-CH_3_); 7.32–7.58 (5H, m, Ph); 11.61 (1H, br.s., NH). ^13^C {^1^H} NMR (101 MHz, CDCl_3_): δ = 14.4; 21.5; 111.5; 128.5; 128.7; 129.9; 131.3; 139.2; 143.0; 144.7; 150.5; 151.1. Anal. Calcd for C_14_H_12_N_4_O_2_: C, 62.68; H, 4.51; N, 20.88. Found: C, 62.75; H, 4.43; N, 20.82.

*3-Etoxycarbonyl-5-methyl-6-nitro-7-phenylpyrazolo[1,5-a]pyrimidine***(5c)**. The reaction was performed according to the general procedure 2, employing 0.30 mmol of heterocycle **4c** and 0.123 + 0.049 g (0.38 + 0.15 mmol) of PhI(OAc)_2_. Pale yellow solid. Yield, 64 mg (65%); mp, 147–150 °C. IR (ATR): 1714 (C=O); 1534, 1364 (NO_2_). ^1^H NMR (400 MHz, CDCl_3_): δ = 1.42 (3H, t, CH_2_-CH_3_, *J =* 7.1 Hz); 2.79 (3H, s, C-5-CH_3_); 4.44 (2H, q, CH_2_-CH_3_, *J =* 7.2, 2.5 Hz); 7.55–7.71 (5H, m, Ph); 8.59 (1H, s, H-2). ^13^C {^1^H} NMR (101 MHz, CDCl_3_): δ = 14.8; 22.4; 61.1; 104.5; 125.5; 129.3; 129.5; 132.6; 137.6; 142.5; 147.2; 150.1; 154.9; 162.2. Anal. Calcd for C_16_H_14_N_4_O_4_: C, 58.89; H, 4.32; N, 17.17. Found: C, 58.94; H, 4.35; N, 17.10.

*3-Cyano-5-methyl-2-methylthio-6-nitro-7-phenylpyrazolo[1,5-a]pyrimidine***(5d)**. The reaction was performed according to the general procedure 2, employing 0.31 mmol of heterocycle **4d** and 0.123 + 0.049 g (0.38 + 0.15 mmol) of PhI(OAc)_2_. Yellow solid. Yield, 39 mg (40%); mp, 168–172 °C. IR (ATR): 2226 (CN); 1551, 1349 (NO_2_). ^1^H NMR (400 MHz, CDCl_3_): δ = 2.55 (3H, s, S-CH_3_); 2.72 (3H, s, C-5-CH_3_); 7.54–7.69 (5H, m, Ph). ^13^C {^1^H} NMR (101 MHz, CDCl_3_): δ = 13.8; 22.1; 83.3; 111.7; 125.1; 129.4 (2C); 132.8; 137.4; 141.9; 150.8; 155.5; 162.4. Anal. Calcd for C_15_H_11_N_5_O_2_S: C, 55.38; H, 3.41; N, 21.53. Found: C, 55.34; H, 4.44; N, 21.49.

*5-Methyl-6-nitro-7-phenyl-1,2,4-triazolo[1,5-a]pyrimidine***(5e)**. The reaction was performed according to the general procedure 2, employing 0.39 mmol of heterocycle **4e** and 0.157 + 0.063 g (0.49 + 0.19 mmol) of PhI(OAc)_2_. Beige solid. Yield, 65 mg (66%); mp, 228–232 °C. IR (ATR): 1537, 1363 (NO_2_). ^1^H NMR (400 MHz, CDCl_3_): δ = 2.80 (3H, s, CH_3_); 7.58–7.72 (5H, m, Ph); 8.54 (1H, s, H-2). ^13^C {^1^H} NMR (101 MHz, CDCl_3_): δ = 22.3; 125.3; 129.2; 129.7; 133.0; 137.8; 143.0; 154.4; 157.8; 158.7. Anal. Calcdfor C_12_H_9_N_5_O_2_: C, 56.47; H, 3.55; N, 27.44. Found: C, 56.53; H, 3.49; N, 27.49.

*2,5-Dimethyl-6-nitro-7-phenyl-1,2,4-triazolo[1,5-a]pyrimidine***(5f)**. The reaction was performed according to the general procedure 2, employing 0.37 mmol of heterocycle **4f** and 0.149 + 0.059 g (0.46 + 0.18 mmol) of PhI(OAc)_2_. Pale orange solid. Yield, 49 mg (50%); mp, 180–183 °C. IR (ATR): 1542, 1368 (NO_2_). ^1^H NMR (400 MHz, CDCl_3_): δ = 2.59 (3H, s, C-2-CH_3_); 2.80 (3H, s, C-5-CH_3_); 7.55–7.70 (5H, m, Ph); 8.54 (1H, s, H-2). ^13^C {^1^H} NMR (101 MHz, CDCl_3_): δ = 15.7; 22.2; 125.6; 129.2; 129.6; 132.8; 137.4; 142.2; 154.9; 157.0; 169.6. Anal. Calcd for C_13_H_11_N_5_O_2_: C, 57.99; H, 4.12; N, 26.01. Found: C, 58.04; H, 4.20; N, 25.93.

*5-Methyl-6-nitro-7-phenyl-2-trifluoromethyl-1,2,4-triazolo[1,5-a]pyrimidine***(5g)**. The reaction was performed according to the general procedure 2, employing 0.31 mmol of heterocycle **4g** and 0.124 + 0.50 g (0.38 + 0.15 mmol) of PhI(OAc)_2_. White yellow solid. Yield, 41 mg (42%); mp, 172–175 °C. IR (ATR): 1548, 1367 (NO_2_). ^1^H NMR (400 MHz, CDCl_3_): δ = 2.83 (3H, s, C-5-CH_3_); 7.59–7.74 (5H, m, Ph). ^13^C {^1^H} NMR (101 MHz, CDCl_3_): δ = 22.5; 119.0 (q, *J =* 271.7 Hz); 124.3; 129.4; 129.8; 133.5; 138.7; 143.6; 154.6, 159.8, 159.9 (q, *J =* 40.5 Hz). Anal. Calcd for C_13_H_8_F_3_N_5_O_2_: C, 48.31; H, 2.49; N, 21.67. Found: C, 48.36; H, 2.52; N, 21.61.

*5-Methyl-2-methylthio-6-nitro-7-phenyl-1,2,4-triazolo[1,5-a]pyrimidine***(5f)**. The reaction was performed according to the general procedure 2, employing 0.33 mmol of heterocycle **4h** and 0.133 + 0.053 g (0.41 + 0.17 mmol) of PhI(OAc)_2_. Beige solid. Yield, 39 mg (40%); mp, 193–198 °C. IR (ATR): 1542, 1369 (NO_2_). ^1^H NMR (400 MHz, CDCl_3_): δ = 2.81 (3H, s, C-5-CH_3_); 3.12 (3H, s, S-CH_3_); 7.56–7.73 (5H, m, Ph). ^13^C {^1^H} NMR (101 MHz, CDCl_3_): δ = 22.5; 40.3; 124.4; 129.4; 129.8; 133.4; 138.3; 143.6; 154.7; 159.5; 173.9. Anal. Calcd for C_13_H_11_N_5_O_2_S: C, 51.82; H, 3.68; N, 23.24. Found: C, 51.91; H, 3.59; N, 23.30.

*2-Azido-6-methyl-5-nitro-4-phenylpyrimidine* **(5i′)**. The reaction was performed according to the general procedure 2, employing 0.39 mmol of heterocycle 4i and 0.156 + 0.062 g (0.48 + 0.19 mmol) of PhI(OAc)_2_. White yellow solid. Yield, 44 mg (45%); mp, 118–120 °C. IR (ATR): 2143 (N_3_); 1519, 1329 (NO_2_). ^1^H NMR (400 MHz, CDCl_3_): δ = 2.59 (3H, s, CH_3_); 7.43–7.70 (5H, m, Ph). ^13^C {^1^H} NMR (101 MHz, CDCl_3_): δ = 21.0; 128.4; 159.5; 132.1; 133.4; 142.2; 160.5; 161.6; 163.4. Anal. Calcd for C_11_H_8_N_6_O_2_: C, 51.56; H, 3.15; N, 32.80. Found: C, 51.50; H, 3.13; N, 32.82.

*6-Nitro-7-phenyl-4,7-dihydropyrazolo[1,5-a]pyrimidine***(6a)**. The reaction was performed according to the general procedure 1, employing 0.166 g (2 mmol, 1 equiv.) of 3-aminopyrazole **1a**, 0.316 g (2 mmol, 1 equiv.) of 1-morpholino-2-nitroethylene **2a**, and 0.20 mL (2 mmol, 1 equiv.) of benzaldehyde **3g**. Yellow solid. Yield, 0.387 g (80%); mp, 289–295 °C. IR (ATR): 1528, 1417 (NO_2_). ^1^H NMR (400 MHz, DMSO-*d*_6_): δ = 5.43 (1H, s, H-7); 7.10–7.30 (5H, m, Ph); 7.41 (1H, s, H-2); 8.36 (1H, d, H-5, *J* = 6.1 Hz); 10.88 (1H, d, NH, *J* = 6.4 Hz); 12.45 (1H, s, H-3). ^13^C {^1^H} NMR (101 MHz, DMSO-*d*_6_): δ = 38.2; 106.8; 124.3; 126.2; 126.5; 128.4; 127.3; 139.0; 144.2; 146.0. Anal. Calcd for C_12_H_10_N_4_O_2_: C, 59.50; H, 4.16; N, 23.13. Found: C, 59.61; H, 4.20; N, 23.01.

*5-Ethyl-6-nitro-7-phenyl-4,7-dihydropyrazolo[1,5-a]pyrimidine***(6b)**. The reaction was performed according to the general procedure 1, employing 0.166 g (2 mmol, 1 equiv.) of 3-aminopyrazole **1a**, 0.372 g (2 mmol, 1 equiv.) of 1-morpholino-2-nitrobuthylene **2c**, and 0.20 mL (2 mmol, 1 equiv.) of benzaldehyde **3g**. Green yellow solid. Yield, 0.243 (45%); mp, 231–234 °C. IR (ATR): 1511, 1426 (NO_2_). ^1^H NMR (400 MHz, DMSO-*d*_6_): δ = 1.29 (3H, t, CH_3_, *J =* 7.2 Hz); 2.90–3.05 (2H, m, CH_2_); 5.48 (1H, s, H-7); 7.09–7.32 (5H, m, Ph); 7.41 (1H, s, H-2); 10.73 (1H, s, NH); 12.37 (1H, s, H-3). ^13^C {^1^H} NMR (101 MHz, DMSO-*d*_6_): δ = 13.0; 27.3; 39.5; 106.8; 122.1; 122.1; 126.0; 126.2; 126.4; 128.3; 144.3; 146.8; 156.4. Anal. Calcd for C_14_H_14_N_4_O_2_: C, 62.21; H, 5.22; N, 20.73. Found: C, 62.28; H, 5.18; N, 20.65.

*6-Nitro-7-phenylpyrazolo[1,5-a]pyrimidine***(7a)**. The reaction was performed according to the general procedure 2, employing 0.41 mmol of heterocycle **6a** and 0.166 + 0.067 g (0.52 + 0.21 mmol) of PhI(OAc)_2_. Light orange solid. Yield, 75 mg (76%); mp, 205–213 °C. IR (ATR): 1523, 1358 (NO_2_). ^1^H NMR (400 MHz, CDCl_3_): δ = 7.46–7.60 (5H, m, Ph); 8.10 (1H, s, H-2); 9.25 (1H, d, H-5); 12.24 (1H, s, H-3). ^13^C {^1^H} NMR (101 MHz, CDCl_3_): δ = 115.6; 128.4; 129.5; 130.4; 132.8; 136.7; 140.1; 141.5; 146.4; 152.1. Anal. Calcd for C_12_H_18_N_4_O_2_: C, 60.00; H, 3.36; N, 23.32. Found: C, 59.89; H, 3.28; N, 23.40.

*5-Ethyl-6-nitro-7-phenylpyrazolo[1,5-a]pyrimidine* **(7b)**. The reaction was performed according to the general procedure 2, employing 0.37 mmol of heterocycle **6b** and 0.149 + 0.060 g (0.46 + 0.19 mmol) of PhI(OAc)_2_. Pale yellow solid. Yield, 69 mg (70%); mp, 198–205 °C. IR (ATR): 1522, 1341 (NO_2_). ^1^H NMR (400 MHz, CDCl_3_): δ = 1.53 (3H, t, CH_3_, *J =* 7.5 Hz); 3.08 (2H, q, CH_2_, *J =* 7.5 Hz); 7.50–7.60 (5H, m, Ph); 8.06 (1H, s, H-2); 12.75 (1H, s, H-3). ^13^C {^1^H} NMR (101 MHz, CDCl_3_): δ = 13.5; 28.0; 113.3; 128.4; 129.7; 130.6; 132.4; 135.6; 138.1; 142.6; 150.8; 156.0. Anal. Calcd for C_14_H_12_N_4_O_2_: C, 62.68; H, 4.51; N, 20.88. Found: C, 62.61; H, 4.54; N, 20.81.

*7-Ethyl-6-nitro-5-methyl-4,7-dihydropyrazolo[1,5-a]pyrimidine***(8a)**. The reaction was performed according to the general procedure 1, employing 0.166 g (2 mmol, 1 equiv.) of 3-aminopyrazole **1a**, 0.344 g (2 mmol, 1 equiv.) of 1-morpholino-2-nitropropylene **2b**, and 0.14 mL (2 mmol, 1 equiv.) of propionaldehyde **3a**. Reaction mixture was heated in a glass autoclave. Orange solid. Yield, 0.175 g (42%); mp, 235 °C with decomp. IR (ATR): 1515, 1278 (NO_2_). ^1^H NMR (400 MHz, DMSO-*d*_6_): δ = 0.72 (3H, t, CH_2_-CH_3_, *J =* 7.4 Hz); 1.45–1.65 (2H, m, CH_2_-CH_3_); 2.52 (3H, s, C-5-CH_3_); 4.23–4.34 (1H, m, H-7); 7.54 (1H, s, H-2); 10.54 (1H, s, NH); 12.36 (1H, s, H-3). ^13^C {^1^H} NMR (101 MHz, DMSO-*d*_6_): δ = 9.8; 21.9; 28.4; 34.4; 105.0; 122.6; 125.9; 145.7; 152.0. Anal. Calcd for C_9_H_12_N_4_O_2_: C, 51.92; H, 5.81; N, 26.91. Found: C, 52.08; H, 5.92; N, 26.75.

*6-Nitro-5-methyl-7-(4ʹ-nitrophenyl)-4,7-dihydropyrazolo[1,5-a]pyrimidine***(8b)**. To a suspension of 0.166 g (2 mmol, 1 equiv.) of 3-aminopyrazole **1a**, 0.344 g (2 mmol, 1 equiv.) of 1-morpholino-2-nitropropylene **2b** in 5 mL of n-BuOH, 3 mmol (1.5 equiv., 0.37 mL) of BF_3_·Et_2_O was added. The reaction mixture was heated on oil bath at 120 °C for 15 min. To the obtained solution, 0.302 g (2 mmol, 1 equiv.) of 4-nitrobenzaldehyde **3b** was added. The reaction mixture was heated on oil bath at 120 °C for 2 h. The resulting solution was cooled to room temperature and stirred for 15 min. The obtained precipitate was filtered off and washed with 15 mL of *i*-PrOH. The precipitate was suspended in 50 mL of water, stirred for 5 min, filtered off again, and washed with 15 mL of water. To the residue, 20 mL of 2 M Na_2_CO_3_ and 50 mL of water were added and stirred for 20 min. The solution was extracted twice with 20 mL of EtOAc. To the water phase, 15 mL of hexane was added, and the mixture was neutralized by diluted HCl to pH 7. The resulting mixture was stirred overnight, filtered off, and washed with water. Yellow solid. Yield, 0.355 g (59%); mp, 197 °C with decomp.

IR (ATR): 1535, 1352 (NO_2_); 1508, 1268 (NO_2_). ^1^H NMR (600 MHz, DMSO-*d*_6_): δ = 2.66 (3H, s, C-5-CH_3_); 5.64 (1H, s, H-5); 7.45 (1H, s, H-2); 7.50 (2H, d, H-2ʹ, *J =* 8.3 Hz); 8.12 (2H, d, H-3ʹ, *J =* 8.3 Hz); 10.95 (1H, s, NH); 12.49 (1H, s, H-3). ^13^C {^1^H} NMR (151 MHz, DMSO-*d*_6_): δ = 22.0; 39.8; 105.6; 121.8; 123.8; 127.0; 127.6; 144.2; 145.9; 152.5; 154.1. Anal. Calcd for C_13_H_11_N_5_O_4_: C, 51.83; H, 3.68; N, 23.25 Found: C, 51.89; H, 3.73; N, 23.19.

*6-Nitro-5-methyl-7-(4ʹ-metoxyphenyl)-4,7-dihydropyrazolo[1,5-a]pyrimidine* **(8c)**. The reaction was performed according to the general procedure 1, employing 0.166 g (2 mmol, 1 equiv.) of 3-aminopyrazole **1a**, 0.344 g (2 mmol, 1 equiv.) of 1-morpholino-2-nitropropylene **2b**, and 0.24 mL (2 mmol, 1 equiv.) of 4-metoxybenzaldehyde **3c**. Yellow solid. Yield, 0.355 (62%); mp, 264–266 °C. IR (ATR): 1509, 1260 (NO_2_). ^1^H NMR (600 MHz, DMSO-*d*_6_): δ = 2.61 (3H, s, C-5-CH_3_); 3.68 (3H, s, O-CH_3_); 5.43 (1H, s, H-5); 6.80 (2H, d, H-3ʹ, *J =* 8.7 Hz); 7.10 (2H, d, H-2ʹ, *J =* 8.7 Hz); 7.39 (1H, s, H-2); 10.72 (1H, s, NH); 12.37 (1H, s, H-3). ^13^C {^1^H} NMR (151 MHz, DMSO-*d*_6_): δ = 22.0; 38.7; 55.0; 107.1; 113.7; 123.3; 126.4; 127.5; 139.0; 144.5; 151.2; 157.6. Anal. Calcd for C_14_H_14_N_4_O_3_: C, 58.74; H, 4.93; N, 19.57. Found: C, 58.78; H, 4.91; N, 19.61.

*6-Nitro-5-methyl-7-(4ʹ-hydroxy-3ʹ-metoxyphenyl)-4,7-dihydropyrazolo[1,5-a]pyrimidine***(8d)**. The reaction was performed according to the general procedure 1, employing 0.166 g (2 mmol, 1 equiv.) of 3-aminopyrazole **1a**, 0.344 g (2 mmol, 1 equiv.) of 1-morpholino-2-nitropropylene **2b**, and 0.24 g (2 mmol, 1 equiv.) of 4-hydroxy-3-metoxybenzaldehyde **3d**. Yellow solid. Yield, 0.314 (52%); mp, 224-226 °C. IR (ATR): 3170 (OH); 1515, 1264 (NO_2_). ^1^H NMR (400 MHz, DMSO-*d*_6_): δ = 2.61 (3H, s, C-5-CH_3_); 3.73 (3H, s, O-CH_3_); 5.39 (1H, s, H-5); 6.50 (1H, dd, H-5ʹ, *J_1_ =* 8.1 Hz, *J_1_ =* 2.1 Hz); 6.62 (1H, d, H-6ʹ, *J =* 8.1 Hz); 6.79 (1H, s, H-2ʹ); 7.43 (1H, s, H-2); 8.68 (1H, s, OH); 10.65 (1H, s, NH); 12.33 (1H, s, H-3). ^13^C {^1^H} NMR (151 MHz, DMSO-*d*_6_): δ = 21.8; 38.9; 55.6; 107.2; 111.0; 115.3; 118.1; 123.0; 126.3; 137.9; 144.3; 144.8; 147.4; 151.1. Anal. Calcd for C_14_H_14_N_4_O_4_: C, 55.63; H, 4.67; N, 18.53. Found: C, 55.69; H, 4.71; N, 18.50.

*6-Nitro-5-methyl-7-(anthracene-9ʹ-yl)-4,7-dihydropyrazolo[1,5-a]pyrimidine* **(8e)**. The reaction was performed according to the general procedure 1, employing 0.166 g (2 mmol, 1 equiv.) of 3-aminopyrazole 1a, 0.344 g (2 mmol, 1 equiv.) of 1-morpholino-2-nitropropylene 2b, and 0.412 g (2 mmol, 1 equiv.) of 9-anthraldehyde 3e. The product was recrystallized from *n-*BuOH. The substance was dried over P_2_O_5_ at 140 °C. Pale green solid. Yield, 0.363 (51%); mp, 222 °C with decomp. IR (ATR): 1522, 1290 (NO_2_). ^1^H NMR (400 MHz, DMSO-*d*_6_): δ = 2.66 (3H, s, C-5-CH_3_); 7.02 (1H, s, H-5); 7.13 (1H, s, H-10ʹ); 7.29–7.40 (2H, m, H-3ʹ, H-6); 7.50–7.67 (2H, m, H-2ʹ, H-7ʹ); 8.03 (1H, d, H-1ʹ, *J =* 8.3 Hz); 8.05–8.15 (2H, m, H-4ʹ, H-5ʹ); 8.48 (1H, s, H-2); 8.79 (1H, d, H-8ʹ, *J =* 9.0 Hz); 11.01 (1H, s, NH); 12.32 (1H, s, H-3). ^13^C {^1^H} NMR (151 MHz, DMSO-*d*_6_): δ = 21.7; 34.1; 106.4; 123.7; 124.1; 124.4; 124.9; 125.0 (2C); 126.3; 126.4; 126.6; 128.2; 128.9; 129.5; 131.0; 131.7; 136.9; 144.7; 150.5. Anal. Calcd for C_21_H_16_N_4_O_2_: C, 70.77; H, 4.53; N, 15.72. Found: C, 70.74; H, 4.51; N, 15.76.

*6-Nitro-5-methyl-7-(thiophen-2ʹ-yl)-4,7-dihydropyrazolo[1,5-a]pyrimidine* **(8f)**. The reaction was performed according to the general procedure 1, employing 0.166 g (2 mmol, 1 equiv.) of 3-aminopyrazole **1a**, 0.344 g (2 mmol, 1 equiv.) of 1-morpholino-2-nitropropylene **2b**, and 0.184 mL (2 mmol, 1 equiv.) of thiophen-2-carbaldehyde **3d**. Pale green solid. Yield, 0.278 (53%); mp, 208 °C with decomp. IR (ATR): 1511, 1256 (NO_2_). ^1^H NMR (400 MHz, DMSO-*d*_6_): δ = 2.58 (3H, s, C-5-CH_3_); 5.84 (1H, s, H-7); 6.80–6.82 (1H, m, H-3ʹ); 6.83–6.87 (1H, m, H-4ʹ); 7.23 (1H, d, H-5ʹ, *J =* 5.0 Hz); 7.56 (1H, s, H-2); 10.85 (1H, s, NH); 12.49 (1H, s, H-3). ^13^C {^1^H} NMR (101 MHz, DMSO-*d*_6_): δ = 21.8; 34.3; 106.2; 122.9; 123.1; 123.6; 126.5; 126.7; 144.5; 150.6; 151.1. Anal. Calcd for C_11_H_10_N_4_O_2_S: C, 50.37; H, 3.84; N, 21.36. Found: C, 50.20; H, 3.99; N, 21.49.

*7-Ethyl-6-nitro-5-methylpyrazolo[1,5-a]pyrimidine***(9a)**. The reaction was performed according to the general procedure 2, employing 0.48 mmol of heterocycle **8a** and 0.194 + 0.077 g (0.60 + 0.24 mmol) of PhI(OAc)_2_. White yellow solid. Yield, 49 mg (50%); mp, 175–179 °C. IR (ATR): 1521, 1360 (NO_2_). ^1^H NMR (400 MHz, CDCl_3_): δ = 1.42 (3H, t, CH_2_-CH_3_, *J =* 7.6 Hz); 2.76 (3H, s, C-5-CH_3_); 1.42 (2H, q, CH_2_-CH_3_, *J =* 7.6 Hz); 8.23 (1H, s, H-2); 13.02 (1H, s, H-3). ^13^C {^1^H} NMR (101 MHz, CDCl_3_): δ = 15.1; 21.9; 23.6; 113.4; 134.5; 141.9; 143.6; 150.5; 151.4. Anal. Calcd for C_9_H_10_N_4_O_2_: C, 52.42; H, 4.89; N, 27.17. Found: C, 52.39; H, 4.89; N, 27.20.

*6-Nitro-5-methyl-7-(4ʹ-nitrophenyl)-pyrazolo[1,5-a]pyrimidine***(9b)**. The reaction was performed according to the general procedure 2, employing 0.33 mmol of heterocycle **8b** and 0.134 + 0.054 g (0.42 + 0.17 mmol) of PhI(OAc)_2_. Pale yellow solid. Yield, 49 mg (50%); mp, 243 °C with decomp. IR (ATR): 1542, 1344 (NO_2_); 1522, 1369 (NO_2_). ^1^H NMR (400 MHz, DMSO-*d*_6_): δ = 2.68 (3H, s, CH_3_); 7.82 (2H, d, H-2ʹ, *J =* 8.6 Hz); 8.09 (1H, s, H-2); 8.41 (2H, d, H-3ʹ, *J =* 8.6 Hz); 14.26 (1H, s, H-3). ^13^C {^1^H} NMR (101 MHz, DMSO-*d*_6_): δ = 21.5; 111.6; 124.3; 129.6; 134.2; 135.0; 138.5; 140.8; 148.3; 150.1; 150.2. Anal. Calcd for C_13_H_9_N_5_O_4_: C, 52.18; H, 3.03; N, 23.40. Found: C, 52.25; H, 2.99; N, 23.38.

6-Nitro-5-methyl-7-(anthracene-9ʹ-yl)-pyrazolo[1,5-a]pyrimidine **(9e)**, 9-acetoxy-10-oxoanthracene (10), anthraquinone (11). The reaction was performed according to the general procedure 2, employing 0.28 mmol of heterocycle **8e** and 0.113 + 0.045 g (0.35 + 0.14 mmol) of PhI(OAc)_2_. *6-Nitro-5-methyl-7-(anthracene-9ʹ-yl)-pyrazolo[1,5-a]pyrimidine* **(9e)**. The substance was dried over P_2_O_5_ at 140 ^o^C. Sand color solid. Yield, 29 mg (30%); mp, 250 °C with decomp. IR (ATR): 1529, 1373 (NO_2_). ^1^H NMR (400 MHz, DMSO-*d*_6_): δ = 2.78 (3H, s, C-5-CH_3_); 7.36 (2H, d, H-4ʹ, H-5ʹ, *J =* 8.8 Hz); 7.40–7.47 (2H, m, H-2ʹ, H-7ʹ); 7.50 (1H, s, H-10ʹ); 7.52–7.58 (2H, m, H-3ʹ, H-6ʹ); 8.21 (2H, d, H-1ʹ, H-8ʹ, *J =* 8.6 Hz); 8.84 (1H, s, H-2); 14.30 (1H, s, H-3). ^13^C {^1^H} NMR (151 MHz, DMSO-*d*_6_): δ = 21.7; 113.8; 124.9 (2C); 125.1; 125.7 (2C); 127.0 (2C); 128.6; 128.8; 128.9 (2C); 129.1; 130.6; 134.3; 134.4; 134.9; 143.4; 150.1; 150.5.Anal. Calcdfor C_21_H_14_N_4_O_2_: C, 71.18; H, 3.98; N, 15.81. Found: C, 71.26; H, 3.93; N, 15.85.

*9-acetoxy-10-oxoanthracene* (10). Yellow crystals. Yield, 25 mg (35%). ^1^H NMR (400 MHz, DMSO-*d*_6_): δ = 2.19 (3H, s, CH_3_); 7.15 (1H, s, CH); 7.56–7.64 (4H, m, H-2ʹ, H-3ʹ, H-6ʹ, H-7ʹ); 7.70–7.76 (2H, m, H-1ʹ, H-8ʹ); 8.15–8.21 (2H, m, H-4ʹ, H-5ʹ). Mass spectrum, *m*/*z* (I_rel_, %): 208 (100) [M]^+^; 180 (80), 152 (79), 126 (14), 90 (10), 76 (62), 63 (16), 50 (20), 39 (3).

*Anthraquinone* (11). Yellow crystals. Yield, 15 mg (25%). ^1^H NMR (400 MHz, DMSO-*d*_6_): δ = 7.88-7.94 (4H, m, H-2ʹ, H-3ʹ, H-6ʹ, H-7ʹ); 8.21–8.26 (4H, m, H-1ʹ, H-4ʹ, H-5ʹ, H-8ʹ). Mass spectrum, *m*/*z* (I_rel_, %): 252 (13) [M]^+^; 210 (100); 193 (43); 181 (14); 165 (33); 152 (16); 139 (6); 82 (18); 69 (9); 43 (15).

*6-Nitro-5-methyl-7-(thiophen-2ʹ-yl)pyrazolo[1,5-a]pyrimidine* **(9f)**. The reaction was performed according to the general procedure 2, employing 0.38 mmol of heterocycle **8f** and 0.154 + 0.061 g (0.48 + 0.19 mmol) of PhI(OAc)_2_. Light orange solid. Yield, 59 mg (60%); mp, 219 °C with decomp. IR (ATR): 1520, 1366 (NO_2_). ^1^H NMR (400 MHz, DMSO-*d*_6_): δ = 2.59 (3H, s, C-5-CH_3_); 5.84 (1H, s, H-5); 7.29–7.34 (1H, m, H-3ʹ); 7.48–7.53 (1H, m, H-4ʹ); 7.98–8.02 (1H, d, H-5ʹ); 8.33 (1H, s, H-2); 14.19 (1H, s, H-3). ^13^C {^1^H} NMR (151 MHz, DMSO-*d*_6_): δ = 20.8; 110.8; 128.5; 128.9; 130.0; 131.2; 131.3; 134.3; 140.4; 149.5; 150.2. Anal. Calcd for C_11_H_8_N_4_O_2_S: C, 50.76; H, 3.10; N, 21.53. Found: C, 50.73; H, 3.05; N, 21.60.

## 4. Conclusions

Thus, we can conclude that 6-nitroazolo[1,5-a]pyrimidines, even containing an electron donating substituent at position seven, are capable of oxidation with the formation of an aromatic structure. On the other hand, during oxidation, it is necessary to take into consideration the features of these structures since the reaction is often complicated by a side process. However, there is no reason to suppose that the same compounds cannot be obtained by any alternative synthetic approach that excludes the destructive nature of key intermediates. Nevertheless, the obtained experimental and theoretical data correlate well with each other, which indicates the possibility of using this set of methods to study the oxidation reactions of this class of organic compounds. 

## Figures and Tables

**Table 1:**
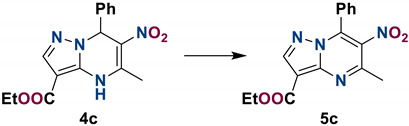
Oxidation of **4c** and reaction parameters.

№	Solvent	Oxidizing Agent	Temperature, °C	Reaction Time, h	Yield, %
1	H_2_O	KMnO_4_, NaOH	25	1	-
2	TFA	O_2_	25	10	-
3	CH_2_Cl_2_	DDQ	25→40	5	-
4	MeCN	DDQ	25→80	5	Trace
5	CH_2_Cl_2_	DMP	25	5	-
6	AcOH	PCC	50	5	49
7	CH_2_Cl_2_	PIDA	25→40	5	-
8	AcOH	PIDA	100	1	81

## Data Availability

Data are contained within article.

## Supplementary Materials

Figures with HOMO distribution, Cyclic voltammograms, and NMR Spectra of compounds **4–11**.

## Sample Availability

Samples of the compounds **4–11** are available from the authors.
