# Supplementary material for: Oxidative Aromatization of 4,7-Dihydro-6-nitroazolo[1,5-a]pyrimidines: Synthetic Possibilities and Limitations, Mechanism of Destruction, and the Theoretical and Experimental Substantiation
Source: Molecules. 2021 Aug 4;26(16):4719. doi: 10.3390/molecules26164719 (PMC8401470; doi:10.3390/molecules26164719)
Supplement: Supplementary file 1 [file molecules-26-04719-s001.zip › molecules-1318306 supplementary/supplementary information/10 and 11.pdf]

Информация о пробе

Analyzed by	: Admin
Analyzed	: 11.06.2021 12:33:44
Sample Type	: Unknown
Level #	: 1
Sample Name	: Ulomsky_L_791_1
Sample ID	: 2998
IS Amount	: [1]=1
Sample Amount	: 1
Dilution Factor	: 1
Vial #	: 1
Injection Volume	: 1.00
Method File	: C:\GCMSsolution\Data\Project1\GH_fast.qgm
Tuning File	: C:\GCMSsolution\System\Tune1\tune 31_05_21.qgt
Modified by	: Admin
Modified	: 11.06.2021 13:03:58

Метод

[Comment]

===== Analytical Line 1 =====

[GCMS-QP2010 Ultra]

IonSourceTemp	:200.00 °C
Interface Temp.	:260.00 °C
Solvent Cut Time	:2.40 min
Detector Gain Mode	:Relative to the Tuning Result
Detector Gain	:+0.00 kV
Threshold	:0

[MS Table]
--Group 1 - Event 1--

Start Time	:2.50min
End Time	:60.00min
ACQ Mode	:Scan
Event Time	:0.25sec
Scan Speed	:2500
Start m/z	:35.00
End m/z	:600.00
Sample Inlet Unit	:GC

Хроматограмма Ulomsky_L_791_1

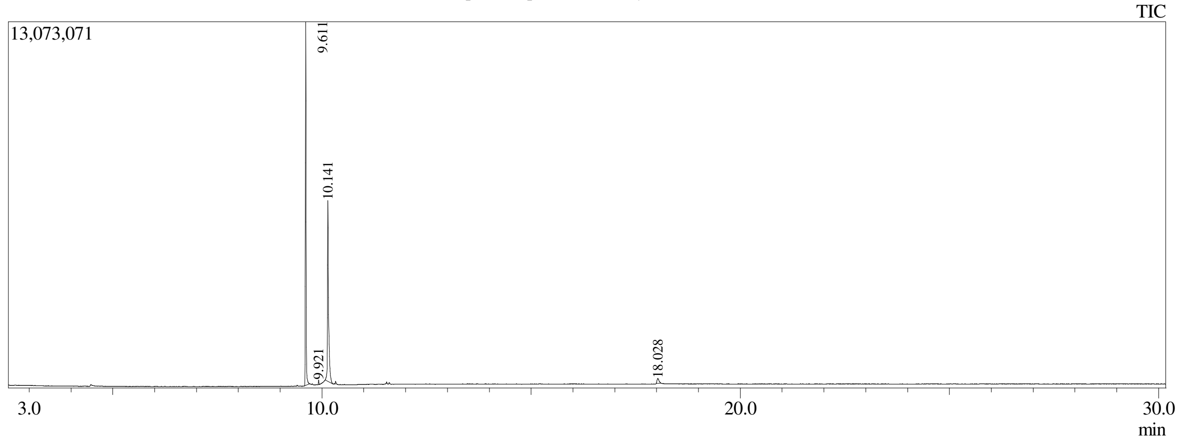

Спектр

Line#:1 R.Time:9.613(Scan#:1708)
MassPeaks:44
RawMode:Averaged 9.608-9.617(1707-1709) BasePeak:208(1874100)
Фон.реж.:Calc. from Peak Group 1 - Event 1

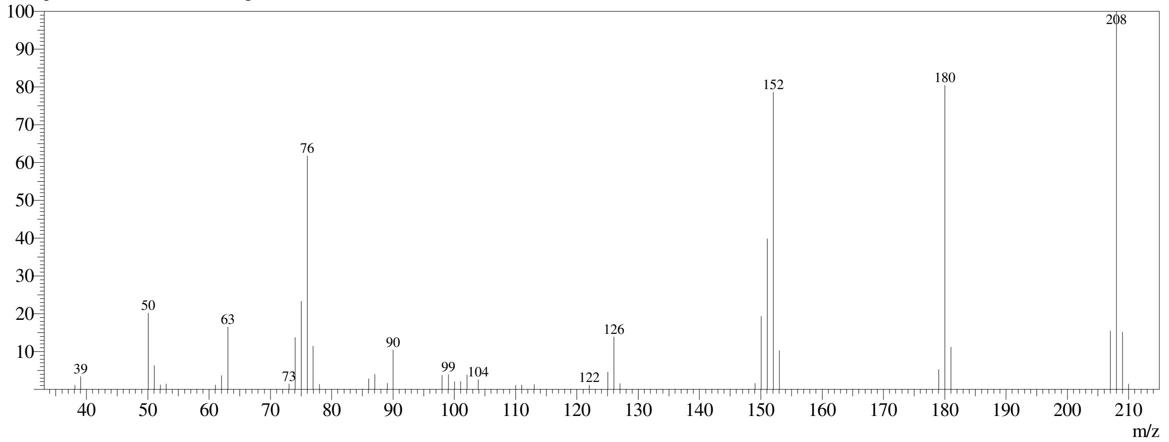

Line#:2   R.Time:9.921(Scan#:1782)
MassPeaks:29
RawMode:Averaged 9.917-9.925(1781-1783) BasePeak:209(42670)
Фон.реж.:Calc. from Peak Group 1 - Event 1

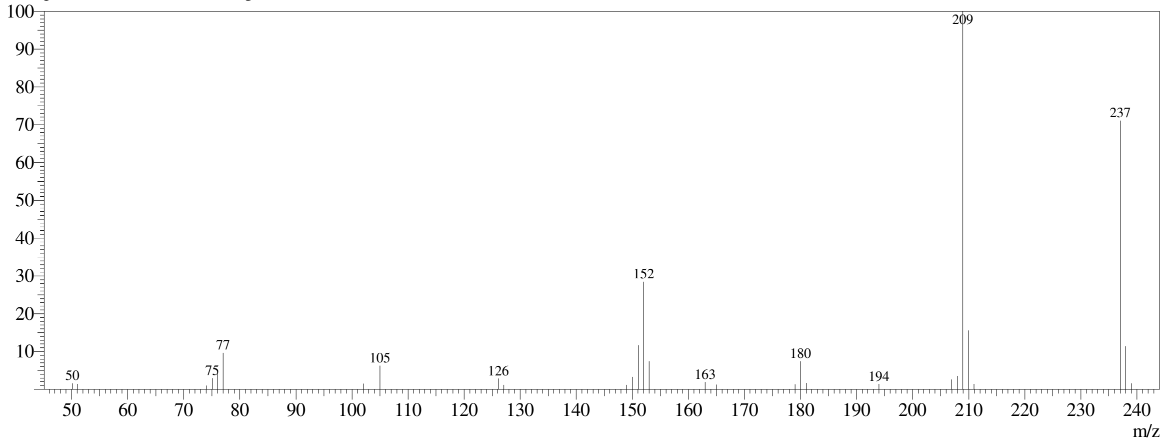

Line#:3   R.Time:10.142(Scan#:1835)
MassPeaks:53
RawMode:Averaged 10.137-10.146(1834-1836) BasePeak:210(1155445)
Фон.реж.:Calc. from Peak Group 1 - Event 1

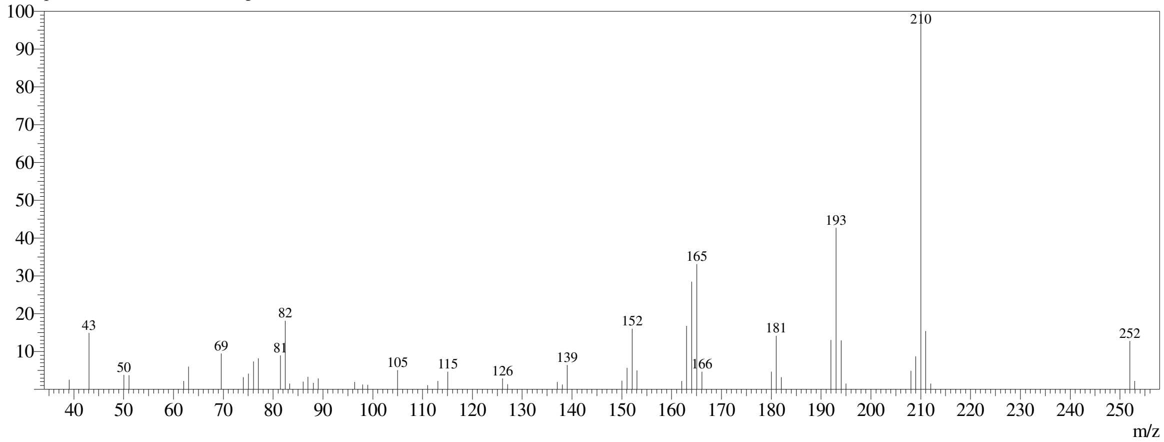

Line#:4   R.Time:18.029(Scan#:3728)
MassPeaks:138
RawMode:Averaged 18.025-18.033(3727-3729) BasePeak:354(28470)
Фон.реж.:Calc. from Peak Group 1 - Event 1

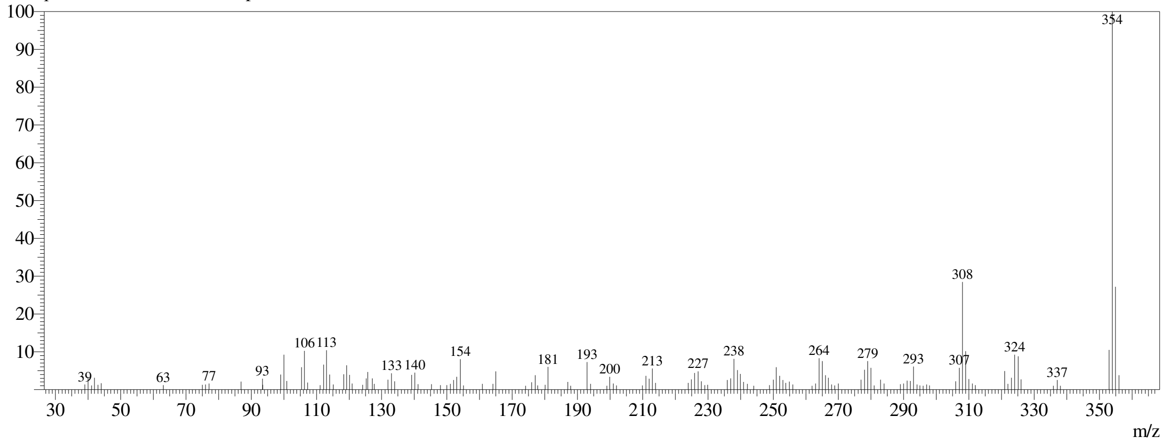

Таблица масс

Line#:1   R.Time:9.613(Scan#:1708)
MassPeaks:44
RawMode:Averaged 9.608-9.617(1707-1709) BasePeak:208(1874100)
BG Mode:Calc. from Peak Group 1 - Event 1

#	m/z	Абс. интен.	Отн. интен.	#	m/z	Абс. интен.	Отн. интен.	#	m/z	Абс. интен.	Отн. интен.
1	38	20916	1.12	2	39	62919	3.36	3	50	378728	20.21

#	m/z	Абс. интен.	Отн. интен.	#	m/z	Абс. интен.	Отн. интен.	#	m/z	Абс. интен.	Отн. интен.
4	51	118022	6.30	18	89	29625	1.58	32	127	28383	1.51
5	52	21782	1.16	19	90	195813	10.45	33	149	29931	1.60
6	53	27767	1.48	20	98	70566	3.77	34	150	362681	19.35
7	61	22732	1.21	21	99	73604	3.93	35	151	746946	39.86
8	62	68471	3.65	22	100	38739	2.07	36	152	1472654	78.58
9	63	309048	16.49	23	101	40099	2.14	37	153	191943	10.24
10	73	26345	1.41	24	102	71085	3.79	38	179	97934	5.23
11	74	256657	13.69	25	104	47575	2.54	39	180	1507042	80.41
12	75	437206	23.33	26	110	21153	1.13	40	181	210177	11.21
13	76	1158586	61.82	27	111	22389	1.19	41	207	288870	15.41
14	77	215117	11.48	28	113	24016	1.28	42	208	1874100	100.00
15	78	25311	1.35	29	122	20208	1.08	43	209	283782	15.14
16	86	52010	2.78	30	125	86362	4.61	44	210	27452	1.46
17	87	74053	3.95	31	126	260698	13.91				

Line#:2 R.Time:9.921(Scan#:1782)
MassPeaks:29
RawMode:Averaged 9.917-9.925(1781-1783) BasePeak:209(42670)
BG Mode:Calc. from Peak Group 1 - Event 1

#	m/z	Абс. интен.	Отн. интен.	#	m/z	Абс. интен.	Отн. интен.	#	m/z	Абс. интен.	Отн. интен.
1	50	686	1.61	11	149	489	1.15	21	194	612	1.43
2	51	628	1.47	12	150	1416	3.32	22	207	1125	2.64
3	74	434	1.02	13	151	4973	11.65	23	208	1505	3.53
4	75	1253	2.94	14	152	12168	28.52	24	209	42670	100.00
5	76	1845	4.32	15	153	3198	7.49	25	210	6659	15.61
6	77	4122	9.66	16	163	828	1.94	26	211	630	1.48
7	102	634	1.49	17	165	554	1.30	27	237	30342	71.11
8	105	2685	6.29	18	179	577	1.35	28	238	4882	11.44
9	126	1232	2.89	19	180	3179	7.45	29	239	673	1.58
10	127	522	1.22	20	181	740	1.73				

Line#:3 R.Time:10.142(Scan#:1835)
MassPeaks:53
RawMode:Averaged 10.137-10.146(1834-1836) BasePeak:210(1155445)
BG Mode:Calc. from Peak Group 1 - Event 1

#	m/z	Абс. интен.	Отн. интен.	#	m/z	Абс. интен.	Отн. интен.	#	m/z	Абс. интен.	Отн. интен.
1	39	29492	2.55	19	96	22482	1.95	37	164	328558	28.44
2	43	172034	14.89	20	98	14632	1.27	38	165	382751	33.13
3	50	43983	3.81	21	99	13631	1.18	39	166	54081	4.68
4	51	43528	3.77	22	105	59232	5.13	40	180	53529	4.63
5	62	25927	2.24	23	111	12473	1.08	41	181	163326	14.14
6	63	69830	6.04	24	113	25192	2.18	42	182	37424	3.24
7	69	109237	9.45	25	115	53635	4.64	43	192	151224	13.09
8	74	36982	3.20	26	126	32856	2.84	44	193	493040	42.67
9	75	48306	4.18	27	127	15721	1.36	45	194	150204	13.00
10	76	85022	7.36	28	137	22775	1.97	46	195	17834	1.54
11	77	94611	8.19	29	138	15176	1.31	47	208	56501	4.89
12	81	103993	9.00	30	139	73962	6.40	48	209	101162	8.76
13	82	209966	18.17	31	150	26344	2.28	49	210	1155445	100.00
14	83	17555	1.52	32	151	65217	5.64	50	211	177992	15.40
15	86	23562	2.04	33	152	185203	16.03	51	212	17402	1.51
16	87	38217	3.31	34	153	57834	5.01	52	252	147649	12.78
17	88	19602	1.70	35	162	25139	2.18	53	253	25587	2.21
18	89	33204	2.87	36	163	193804	16.77				

Line#:4 R.Time:18.029(Scan#:3728)
MassPeaks:138
RawMode:Averaged 18.025-18.033(3727-3729) BasePeak:354(28470)
BG Mode:Calc. from Peak Group 1 - Event 1

#	m/z	Абс. интен.	Отн. интен.	#	m/z	Абс. интен.	Отн. интен.	#	m/z	Абс. интен.	Отн. интен.
1	39	384	1.35	28	121	468	1.64	55	180	370	1.30
2	40	883	3.10	29	124	367	1.29	56	181	1722	6.05
3	41	312	1.10	30	125	856	3.01	57	187	579	2.03
4	42	885	3.11	31	126	1334	4.69	58	188	288	1.01
5	43	356	1.25	32	127	841	2.95	59	193	2074	7.28
6	44	484	1.70	33	128	438	1.54	60	194	423	1.49
7	63	326	1.15	34	132	755	2.65	61	199	294	1.03
8	75	361	1.27	35	133	1224	4.30	62	200	963	3.38
9	76	394	1.38	36	134	635	2.23	63	201	458	1.61
10	77	466	1.64	37	139	1115	3.92	64	202	314	1.10
11	87	604	2.12	38	140	1281	4.50	65	210	305	1.07
12	93	815	2.86	39	141	403	1.42	66	211	1037	3.64
13	94	365	1.28	40	145	413	1.45	67	212	822	2.89
14	99	1122	3.94	41	148	330	1.16	68	213	1596	5.61
15	100	2619	9.20	42	150	346	1.22	69	214	518	1.82
16	101	651	2.29	43	151	441	1.55	70	224	507	1.78
17	105	1691	5.94	44	152	714	2.51	71	225	764	2.68
18	106	2921	10.26	45	153	974	3.42	72	226	1258	4.42
19	107	528	1.85	46	154	2295	8.06	73	227	1365	4.79
20	111	326	1.15	47	155	310	1.09	74	228	629	2.21
21	112	1893	6.65	48	161	424	1.49	75	229	331	1.16
22	113	2958	10.39	49	164	424	1.49	76	230	360	1.26
23	114	1140	4.00	50	165	1373	4.82	77	236	715	2.51
24	115	394	1.38	51	174	297	1.04	78	237	839	2.95
25	118	1161	4.08	52	176	552	1.94	79	238	2327	8.17
26	119	1839	6.46	53	177	1087	3.82	80	239	1481	5.20
27	120	1113	3.91	54	178	318	1.12	81	240	1192	4.19

#	m/z	Абс. интен.	Отн. интен.	#	m/z	Абс. интен.	Отн. интен.	#	m/z	Абс. интен.	Отн. интен.
82	241	585	2.05	101	270	467	1.64	120	307	1649	5.79
83	242	430	1.51	102	277	758	2.66	121	308	8112	28.49
84	244	294	1.03	103	278	1497	5.26	122	309	2885	10.13
85	249	334	1.17	104	279	2139	7.51	123	310	789	2.77
86	250	738	2.59	105	280	1639	5.76	124	311	456	1.60
87	251	1696	5.96	106	281	311	1.09	125	312	344	1.21
88	252	1030	3.62	107	283	759	2.67	126	321	1389	4.88
89	253	714	2.51	108	284	450	1.58	127	322	428	1.50
90	254	498	1.75	109	289	406	1.43	128	323	899	3.16
91	255	605	2.13	110	290	457	1.61	129	324	2633	9.25
92	256	377	1.32	111	291	667	2.34	130	325	2507	8.81
93	262	294	1.03	112	292	663	2.33	131	326	770	2.70
94	263	468	1.64	113	293	1742	6.12	132	336	287	1.01
95	264	2369	8.32	114	294	391	1.37	133	337	717	2.52
96	265	2145	7.53	115	295	321	1.13	134	338	288	1.01
97	266	1081	3.80	116	296	291	1.02	135	353	2993	10.51
98	267	897	3.15	117	297	393	1.38	136	354	28470	100.00
99	268	392	1.38	118	298	307	1.08	137	355	7754	27.24
100	269	308	1.08	119	306	630	2.21	138	356	1085	3.81
