# Supplementary material for: Oxidative Aromatization of 4,7-Dihydro-6-nitroazolo[1,5-a]pyrimidines: Synthetic Possibilities and Limitations, Mechanism of Destruction, and the Theoretical and Experimental Substantiation
Source: Molecules. 2021 Aug 4;26(16):4719. doi: 10.3390/molecules26164719 (PMC8401470; doi:10.3390/molecules26164719)
Supplement: Supplementary file 1 [file molecules-26-04719-s001.zip › molecules-1318306 supplementary/supplementary information/supporting information.docx]

Supporting Information for

Oxidative aromatization of 4,7-dihydro-6-nitroazolo[1,5-a]pyrimidines: synthetic possibilities and limitations, mechanism of destruction, and the theoretical and experimental substantiation

Daniil N. Lyapustin.^1^, Evgeny N. Ulomsky ^1,2^, Ilya A. Balyakin ^3,4^, Alexander V. Shchepochkin ^2^, Vladimir L. Rusinov ^1,2,^*, Oleg N. Chupakhin^1,2^

^1^	Department of Organic and Biomolecular Chemistry, Ural Federal University, Mira St. 19, 620002 Ekaterinburg, Russia, [lyapustin.danil@yandex.ru](javascript:void(0);); [ulomsky@yandex.ru](javascript:void(0);)

^2^	Institute of Organic Synthesis, Ural Branch of the Russian Academy of Sciences, S. Kovalevskaya Str., 22, 620041, Ekaterinburg, Russia, [sasha-mmm@mail.ru](javascript:void(0);)

^3^	NANOTECH Centre, Ural Federal University, 620002, Ekaterinburg, Russia,i.a.balyakin@gmail.com

^4^	Institute of Metallurgy, Ural Branch of the Russian Academy of Sciences, 620016, Ekaterinburg, Russia

*	Correspondence: v.l.rusinov@urfu.ru

------------------------------------------------------------------------

Table of Contents:

Computational Details	S-2

Cyclic voltammograms	S-6

NMR Spectra of Compounds **4-11**	S-12

MS of Compounds **10,11**	S-82

Computational Details

Density functional theory (DFT) simulation via Vienna Ab initio Simulation Package (VASP) was performed for structural optimization of the molecules and obtaining of the charge distribution. Length of supercell of every molecule was set to 22.5 Å and cut-off energy of plane wave basis set was set to 750 eV. Structural optimization was performed using generalized gradient approximation (GGA) by Pedrew, Burke and Ernzerhof (PBE) for exchange-correlation potential. For all chemical species projected augmented wave (PAW) pseudopotentials were used. The structures were optimized by two algorithms: conjugate gradient algorithm was implemented for initial optimization and after that RMM-DIIS (residual minimization scheme, direct inversion in the iterative subspace) algorithm was used for final optimization. The stopping criteria for optimization was |𝐹𝑖|≤0.01 𝑒𝑉/Å where i = x, y, z, i.e. absolute value of each force component acting on nuclei should be not greater than 10 meV/Å.

For PBE-optimized structures a hybrid PBE0 simulation was performed to obtain charge distribution. Since all the performed calculation were spin polarized it was possible to extract spin density from the charge distribution. For interpretation of obtained data, the program VESTA^51^ was used.

|  |  |
| --- | --- |
| **4a** | **8a** |

|  |  |
| --- | --- |
| **8b** | **8c** |

|  |  |
| --- | --- |
| **8d** | **8e** |

CYCLIC VOLTAMMOGRAMS

Cyclic voltammetry was carried out on a Metrohm Autolab PGSTAT**128N** potentiostat with a standard three-electrode configuration. Typically, a three electrodes cell equipped with a glass carbon working electrode, a Ag/AgNO_3_ (0.01M) reference electrode, and a glass carbon rod counter electrode was employed. The measurements were done in acetonitrile with tetrabutylammonium hexafluorophosphate (0.1 M) as the supporting electrolyte under an argon atmosphere at a scan rate of 100 mV/s. C_(_**_4a,8a-e)_** = 5$\cdot$10^-3^ M.

**5-Methyl-6-nitro-7-phenyl-4,7-dihydropyrazolo[1,5-a]pyrimidine** **4a, ^1^Н NMR, 400 MHz, DMSO-*d_6_***

**5-Methyl-6-nitro-7-phenyl-4,7-dihydropyrazolo[1,5-a]pyrimidine** **4a, ^1^Н NMR, 101 MHz, DMSO-*d _6_***

**5-Methyl-6-nitro-7-phenyl-4,7-dihydropyrazolo[1,5-a]pyrimidine** **4a, ^1^Н-^13^C HMBC, 400/101 Mhz, DMSO-*d _6_***

**2,5-Dimethyl-6-nitro-7-phenyl-4,7-dihydropyrazolo[1,5-a]pyrimidine** **4b, ^1^Н NMR, 400 MHz, DMSO-*d_6_***

**2,5-Dimethyl-6-nitro-7-phenyl-4,7-dihydropyrazolo[1,5-a]pyrimidine** **4b, ^13^C NMR, 101 MHz, DMSO-*d_6_***

**3-Etoxycarbonyl-5-Methyl-6-nitro-7-phenyl-4,7-dihydropyrazolo[1,5-a]pyrimidine** **4c, ^1^Н NMR, 400 MHz, DMSO-*d_6_***

**3-Etoxycarbonyl-5-Methyl-6-nitro-7-phenyl-4,7-dihydropyrazolo[1,5-a]pyrimidine** **4c, ^13^C NMR, 101 MHz, DMSO-*d_6_***

**3-Cyano-5-methyl-2-methylthio-6-nitro-7-phenyl-4,7-dihydropyrazolo[1,5-a]pyrimidine 4d, ^1^Н NMR, 400 MHz, DMSO-*d_6_***

**3-Cyano-5-methyl-2-methylthio-6-nitro-7-phenyl-4,7-dihydropyrazolo[1,5-a]pyrimidine 4d, ^13^C NMR, 101 MHz, DMSO-*d_6_***

**5-Methyl-6-nitro-7-phenyl-4,7-dihydro-1,2,4-triazolo[1,5-a]pyrimidine 4e, ^1^Н NMR, 400 MHz, DMSO-*d_6_***

**5-Methyl-6-nitro-7-phenyl-4,7-dihydro-1,2,4-triazolo[1,5-a]pyrimidine 4e, ^13^C NMR, 101 MHz, DMSO-*d_6_***

**2,5-Dimethyl-6-nitro-7-phenyl-4,7-dihydro-1,2,4-triazolo[1,5-a]pyrimidine 4f, ^1^Н NMR, 400 MHz, DMSO-*d_6_***

**2,5-Dimethyl-6-nitro-7-phenyl-4,7-dihydro-1,2,4-triazolo[1,5-a]pyrimidine 4f, ^13^C NMR, 101 MHz, DMSO-*d_6_***

**5-Methyl-6-nitro-7-phenyl-2-trifluoromethyl-4,7-dihydro-1,2,4-triazolo[1,5-a]pyrimidine** **4g, ^1^Н NMR, 400 MHz, DMSO-*d_6_***

**5-Methyl-6-nitro-7-phenyl-2-trifluoromethyl-4,7-dihydro-1,2,4-triazolo[1,5-a]pyrimidine** **4g, ^13^C NMR, 101 MHz, DMSO-*d_6_***

**5-Methyl-2-methylthio-6-nitro-7-phenyl-4,7-dihydro-1,2,4-triazolo[1,5-a]pyrimidine 4h, ^1^Н NMR, 400 MHz, DMSO-*d_6_***

**5-Methyl-2-methylthio-6-nitro-7-phenyl-4,7-dihydro-1,2,4-triazolo[1,5-a]pyrimidine 4h, ^13^C NMR, 101 MHz, DMSO-*d_6_***

**5-Methyl-6-nitro-7-phenyl-4,7-dihydrotetrazolo[1,5-a]pyrimidine** **4i, ^1^Н NMR, 400 MHz, DMSO-*d_6_***

**5-Methyl-6-nitro-7-phenyl-4,7-dihydrotetrazolo[1,5-a]pyrimidine** **4i, ^13^C NMR, 101 MHz, DMSO-*d_6_***

**5-Methyl-6-nitro-7-phenylpyrazolo[1,5-a]pyrimidine** **5a, ^1^Н NMR, 400 MHz, CDCl_3_**

**5-Methyl-6-nitro-7-phenylpyrazolo[1,5-a]pyrimidine** **5a, ^13^C NMR, 101 MHz, CDCl_3_**

**5-Dimethyl-6-nitro-7-phenylpyrazolo[1,5-a]pyrimidine** **5b, ^1^Н NMR, 400 MHz, CDCl_3_**

**5-Dimethyl-6-nitro-7-phenylpyrazolo[1,5-a]pyrimidine** **5a, ^13^C NMR, 101 MHz, CDCl_3_**

**3-Etoxycarbonyl-5-methyl-6-nitro-7-phenylpyrazolo[1,5-a]pyrimidine 5c, ^1^Н NMR, 400 MHz, CDCl_3_**

**3-Etoxycarbonyl-5-methyl-6-nitro-7-phenylpyrazolo[1,5-a]pyrimidine 5c, ^13^C NMR, 101 MHz, CDCl_3_**

**3-Cyano-5-methyl-2-methylthio-6-nitro-7-phenylpyrazolo[1,5-a]pyrimidine 5d, ^1^Н NMR, 400 MHz, CDCl_3_**

**3-Cyano-5-methyl-2-methylthio-6-nitro-7-phenylpyrazolo[1,5-a]pyrimidine 5d, ^13^C NMR, 101 MHz, CDCl_3_**

**5-Methyl-6-nitro-7-phenyl-1,2,4-triazolo[1,5-a]pyrimidine 5e, ^1^Н NMR, 400 MHz, CDCl_3_**

**5-Methyl-6-nitro-7-phenyl-1,2,4-triazolo[1,5-a]pyrimidine 5e, ^13^C NMR, 101 MHz, CDCl_3_**

**5-Dimethyl-6-nitro-7-phenyl-1,2,4-triazolo[1,5-a]pyrimidine 5f, ^1^Н NMR, 400 MHz, CDCl_3_**

**5-Dimethyl-6-nitro-7-phenyl-1,2,4-triazolo[1,5-a]pyrimidine 5f, ^13^C NMR, 101 MHz, CDCl_3_**

**5-Methyl-6-nitro-7-phenyl-2-trifluoromethyl-1,2,4-triazolo[1,5-a]pyrimidine 5g, ^1^Н NMR, 400 MHz, CDCl_3_**

**5-Methyl-6-nitro-7-phenyl-2-trifluoromethyl-1,2,4-triazolo[1,5-a]pyrimidine 5g, ^13^C NMR, 101 MHz, CDCl_3_**

**5-Methyl-2-methylthio-6-nitro-7-phenyl-1,2,4-triazolo[1,5-a]pyrimidine 5h, ^1^Н NMR, 400 MHz, CDCl_3_**

**5-Methyl-2-methylthio-6-nitro-7-phenyl-1,2,4-triazolo[1,5-a]pyrimidine 5h, ^13^C NMR, 101 MHz, CDCl_3_**

**2-Azido-6-methyl-5-nitro-4-phenylpyrimidine 5iʹ, ^1^Н NMR, 400 MHz, CDCl_3_**

**2-Azido-6-methyl-5-nitro-4-phenylpyrimidine 5iʹ, ^13^C NMR, 101 MHz, CDCl_3_**

**6-Nitro-7-phenyl-4,7-dihydropyrazolo[1,5-a]pyrimidine 6a, ^1^Н NMR, 400 MHz,DMSO-*d*_6_**

**6-Nitro-7-phenyl-4,7-dihydropyrazolo[1,5-a]pyrimidine 6a, ^13^C NMR, 101 MHz,DMSO-*d*_6_**

**5-Ethyl-6-nitro-7-phenyl-4,7-dihydropyrazolo[1,5-a]pyrimidine 6b, ^1^Н NMR, 400 MHz,DMSO-*d*_6_**

**5-Ethyl-6-nitro-7-phenyl-4,7-dihydropyrazolo[1,5-a]pyrimidine 6b, ^13^C NMR, 101 MHz,DMSO-*d*_6_**

**5-Ethyl-6-nitro-7-phenyl-4,7-dihydropyrazolo[1,5-a]pyrimidine 6b, ^1^H-^13^C HMBC NMR, 400/101 MHz,DMSO-*d*_6_**

**6-Nitro-7-phenylpyrazolo[1,5-a]pyrimidine 7a, ^1^Н NMR, 400 MHz, CDCl_3_**

**6-Nitro-7-phenylpyrazolo[1,5-a]pyrimidine 7a, ^13^C NMR, 101 MHz, CDCl_3_**

**5-Ethyl-6-nitro-7-phenylpyrazolo[1,5-a]pyrimidine 7b, ^1^Н NMR, 400 MHz, CDCl_3_**

**5-Ethyl-6-nitro-7-phenylpyrazolo[1,5-a]pyrimidine 7b, ^13^C NMR, 101 MHz, CDCl_3_**

**7-Ethyl-6-nitro-5-methyl-4,7-dihydropyrazolo[1,5-a]pyrimidine** **8a, ^1^Н NMR, 400 MHz, DMSO-*d*_6_**

**7-Ethyl-6-nitro-5-methyl-4,7-dihydropyrazolo[1,5-a]pyrimidine** **8a, ^13^C NMR, 101 MHz, DMSO-*d*_6_**

**6-Nitro-5-methyl-7-(4ʹ-nitrophenyl)-4,7-dihydropyrazolo[1,5-a]pyrimidine 8b, ^1^Н NMR, 600 MHz, DMSO-*d*_6_**

**6-Nitro-5-methyl-7-(4ʹ-nitrophenyl)-4,7-dihydropyrazolo[1,5-a]pyrimidine 8b, ^13^C NMR, 151 MHz, DMSO-*d*_6_**

**6-Nitro-5-methyl-7-(4ʹ-metoxyphenyl)-4,7-dihydropyrazolo[1,5-a]pyrimidine** **8c, ^1^Н NMR, 600 MHz, DMSO-*d*_6_**

**6-Nitro-5-methyl-7-(4ʹ-metoxyphenyl)-4,7-dihydropyrazolo[1,5-a]pyrimidine** **8c, ^13^C NMR, 151 MHz, DMSO-*d*_6_**

**6-Nitro-5-methyl-7-(4ʹ-hydroxy-3ʹ-metoxyphenyl)-4,7-dihydropyrazolo[1,5-a]pyrimidine** **8d, ^1^Н NMR, 400 MHz, DMSO-*d*_6_**

**6-Nitro-5-methyl-7-(4ʹ-hydroxy-3ʹ-metoxyphenyl)-4,7-dihydropyrazolo[1,5-a]pyrimidine** **8d, ^13^C NMR, 101 MHz, DMSO-*d*_6_**

**6-Nitro-5-methyl-7-(anthracene-9ʹ-yl)-4,7-dihydropyrazolo[1,5-a]pyrimidine 8e, ^1^Н NMR, 400 MHz, DMSO-*d*_6_**

**6-Nitro-5-methyl-7-(anthracene-9ʹ-yl)-4,7-dihydropyrazolo[1,5-a]pyrimidine 8e, ^13^C NMR, 101 MHz, DMSO-*d*_6_**

**6-Nitro-5-methyl-7-(thiophen-2ʹ-yl)-4,7-dihydropyrazolo[1,5-a]pyrimidine** **8f, ^1^Н NMR, 400 MHz, DMSO-*d*_6_**

**6-Nitro-5-methyl-7-(thiophen-2ʹ-yl)-4,7-dihydropyrazolo[1,5-a]pyrimidine** **8f, ^13^C NMR, 101 MHz, DMSO-*d*_6_**

**7-Ethyl-6-nitro-5-methylpyrazolo[1,5-a]pyrimidine 9a, ^1^Н NMR, 400 MHz, CDCl_3_**

**7-Ethyl-6-nitro-5-methylpyrazolo[1,5-a]pyrimidine 9a, ^13^C NMR, 101 MHz, CDCl_3_**

**6-Nitro-5-methyl-7-(4ʹ-nitrophenyl)-pyrazolo[1,5-a]pyrimidine 9b, ^1^Н NMR, 400 MHz, DMSO-*d*_6_**

**6-Nitro-5-methyl-7-(4ʹ-nitrophenyl)-pyrazolo[1,5-a]pyrimidine 9b, ^13^C NMR, 101 MHz, DMSO-*d*_6_**

**6-Nitro-5-methyl-7-(4ʹ-metoxyphenyl)-pyrazolo[1,5-a]pyrimidine 9c, ^1^Н NMR, 400 MHz, DMSO-*d*_6_**

**6-Nitro-5-methyl-7-(4ʹ-metoxyphenyl)-pyrazolo[1,5-a]pyrimidine 9c, ^13^C NMR, 101 MHz, DMSO-*d*_6_**

**6-Nitro-5-methyl-7-(anthracene-9ʹ-yl)-pyrazolo[1,5-a]pyrimidine** **9e, ^1^Н NMR, 400 MHz, DMSO-*d*_6_**

**6-Nitro-5-methyl-7-(anthracene-9ʹ-yl)-pyrazolo[1,5-a]pyrimidine** **9e, ^13^C NMR, 101 MHz, DMSO-*d*_6_**

**6-Nitro-5-methyl-7-(thiophen-2ʹ-yl)pyrazolo[1,5-a]pyrimidine** **9f, ^1^Н NMR, 400 MHz, DMSO-*d*_6_**

**6-Nitro-5-methyl-7-(thiophen-2ʹ-yl)pyrazolo[1,5-a]pyrimidine** **9f, ^13^C NMR, 101 MHz, DMSO-*d*_6_**

**9-acetoxy-10-oxoanthracene (10) and anthraquinone (11); ^1^Н NMR, 400 MHz, DMSO-*d*_6_**

**9-acetoxy-10-oxoanthracene (10) and anthraquinone (11); ^1^Н NMR, 400 MHz, DMSO-*d*_6_**

**Mass-spectrum of 9-acetoxy-10-oxoanthracene (10) and anthraquinone (11) (EI, 70 eV)**
